# Rab26 restricts insulin secretion via sequestering Synaptotagmin-1

**DOI:** 10.1371/journal.pbio.3002142

**Published:** 2023-06-08

**Authors:** Ruijuan Zhuang, Yuxia Zhou, Ziyan Wang, Yating Cao, Jun Chen, Liju Xu, Yandan Ren, Yige Zheng, Ziheng Wei, Hantian Qiu, Liangcheng Li, Yang Han, Ye Yun, Xin Chen, Wanjin Hong, Tuanlao Wang

**Affiliations:** 1 School of Pharmaceutical Sciences, State Key Laboratory of Cellular Stress Biology, Fujian Provincial Key Laboratory of Innovative Drug Target Research, Xiamen University, Fujian, China; 2 School of Basic Medical Sciences, Guizhou Provincial Key Laboratory of Pathogenesis and Drug Research on Common Chronic Diseases, Guizhou Medical University, Guiyang, China; 3 State Key Laboratory of Cellular Stress Biology, Innovation Center for Cell Biology, School of Life Sciences, Xiamen University, Fujian, China; 4 Institute of Molecular and Cell Biology, A STAR (Agency of Science, Technology and Research), Singapore, Singapore; Columbia University, UNITED STATES

## Abstract

Rab26 is known to regulate multiple membrane trafficking events, but its role in insulin secretion in pancreatic β cells remains unclear despite it was first identified in the pancreas. In this study, we generated Rab26^-/-^ mice through CRISPR/Cas9 technique. Surprisingly, insulin levels in the blood of the Rab26^-/-^ mice do not decrease upon glucose stimulation but conversely increase. Deficiency of Rab26 promotes insulin secretion, which was independently verified by Rab26 knockdown in pancreatic insulinoma cells. Conversely, overexpression of Rab26 suppresses insulin secretion in both insulinoma cell lines and isolated mouse islets. Islets overexpressing Rab26, upon transplantation, also failed to restore glucose homeostasis in type 1 diabetic mice. Immunofluorescence microscopy revealed that overexpression of Rab26 results in clustering of insulin granules. GST-pulldown experiments reveal that Rab26 interacts with synaptotagmin-1 (Syt1) through directly binding to its C2A domain, which interfering with the interaction between Syt1 and SNAP25, and consequently inhibiting the exocytosis of newcomer insulin granules revealed by TIRF microscopy. Our results suggest that Rab26 serves as a negative regulator of insulin secretion, via suppressing insulin granule fusion with plasma membrane through sequestering Syt1.

## Abstract 2

Despite being first identified in the pancreas, the role of the small GTPase Rab26 in insulin secretion is unclear. This study shows that Rab26 restricts insulin secretion by interacting with Syntaptotagmin-1 to interfere with the fusion of insulin granules with the plasma membrane.

## Introduction

Insulin is the most important hormone to maintain glucose homeostasis. Defect in insulin secretion is the main pathophysiological mechanism accounting for diabetes mellitus [1]. Islet β cells, a small number of endocrine cells comprising less than 2% of the human pancreas in adults [2], secrete insulin in a biphasic manner in response to secretagogues [3]. Insulin secretion includes a series of vesicular trafficking processes tightly regulated by multiple membrane trafficking machineries such as Rab small GTPases, SNARE proteins, and SNARE associated partners. Reduced insulin secretion could be due to either defects in insulin granule transport (granule maturation), docking to or fusion with the plasma membrane, or abnormal degradation of insulin/proinsulin [3–5]. Dysfunction in mitochondria with loss of ATP also results in the impaired glucose-stimulated insulin secretion [6].

Rab small GTPases associate with different subcellular compartments of the exocytotic and endocytic pathways [7]. Rab proteins are the key regulators for vesicular trafficking via serving as the molecular switches cycling between GDP-bound and GTP-bound form [8,9]. GTP-bound form generally engages downstream effectors to regulate vesicle formation and/or tethering or docking. The biogenesis of insulin granules (dense core granules) and insulin secretion are regulated by Rab proteins and their effectors. Two Rab proteins, Rab3 and Rab27, are associated with insulin granules, which interact with multiple effectors to regulate insulin secretion through mediating granule trafficking, docking to the plasma membrane [10]. Especially, mutation in Rab27 caused diabetes in mice [11]. Rab26 was firstly characterized from rat pancreas [12]; however, its role in pancreatic β cells remain unclear.

Several studies revealed that Rab26 plays important roles in regulating protein transport. Rab26 is involved in autophagic pathway by regulating autophagic degradation of phosphorylated Src and also linking synaptic vesicles to the autophagy pathway [13,14]. Rab26 modulates the transport of α2-adrenergic receptors from the Golgi to the cell surface [15]. Rab26 may also regulate maturation of exocrine granules and may be involved in the recruitment of secretory granules to the plasma membrane to mediate amylase release in rat parotid acinar cells [16]. These studies suggest a potential role of Rab26 in regulating other secretory events such as insulin secretion.

In this study employing Rab26 gene knockout mice, we found that deficiency of Rab26 enhances insulin secretion. Conversely, overexpression of Rab26 inhibits insulin secretion in pancreatic insulinoma cells and freshly isolated mouse islets. Rab26 overexpression induces clustering of insulin granules. Mechanistically, Rab26 interacts with Synaptotagmin-1 (Syt1), and this interaction may competitively inhibit Syt1 binding to the SNARE complex to interfere insulin granule fusion to plasma membrane.

## Results

### Deficiency of Rab26 enhances insulin secretion

**Fig 1:**
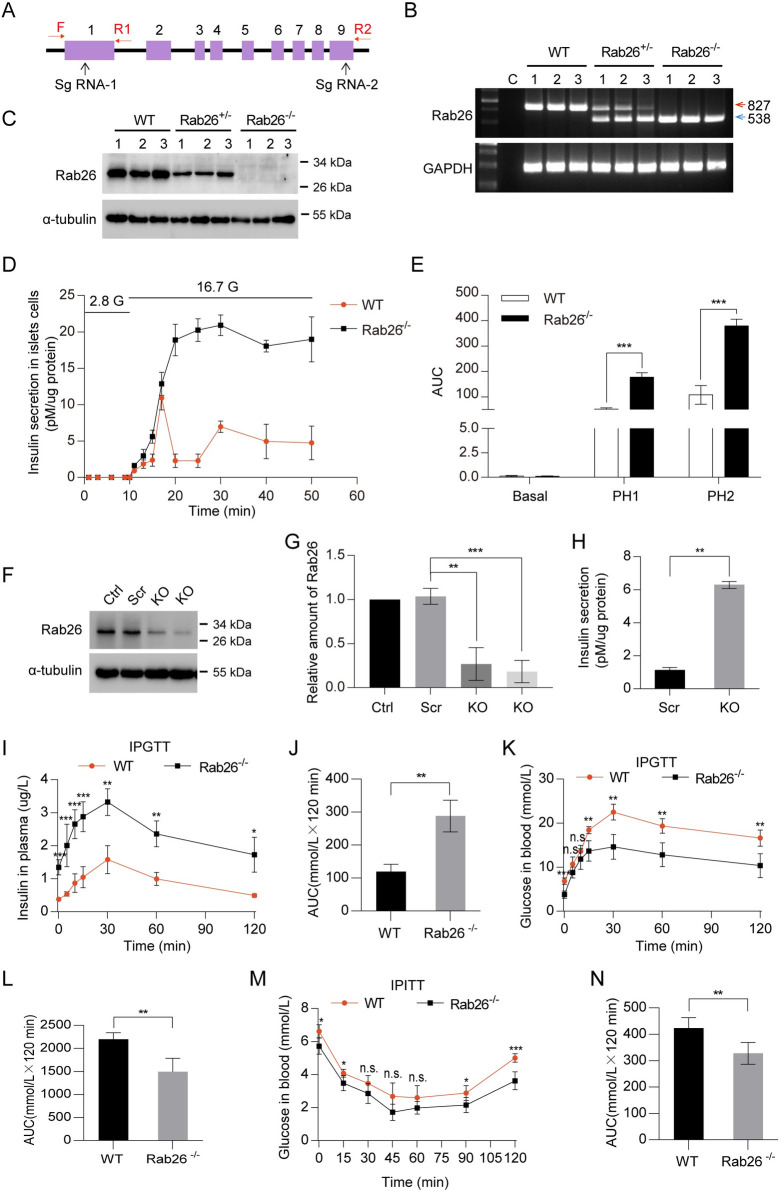
Deficiency of Rab26 enhances insulin secretion. (A) Gene-trap strategy for generating Rab26 KO mice, the location of sg-RNA used for gene KO and the primers genotyping were indicated. (B) PCR products were used to identify mouse tail DNA to confirm Rab26 gene deletion. WTs exhibit 827 bp fragments, heterozygous mutant mice produced 827 bp and 538 bp fragments, and homozygous mutant mice produced 538 bp fragments from genomic DNA, respectively. (C) Western blot using lysates of pancreas isolated from 8-wk-old WT, Rab26^+/−^ and Rab26^−/−^ mice showed the depletion of Rab26. α-tubulin was used as a loading control. (D) A total of 100 fresh mouse islets with uniform size were isolated and cultured in 1640 medium containing 10% FBS. The insulin secretion of mouse islets was detected by ELISA at the appointed time. (E) AUC were calculated. (F) The Rab26 KO INS-1 cell lines were generated by CRISPR/Cas9 technique; western blot detection showed the depletion of Rab26. (G) Quantitative data of F from 3 independent experiments. (H) Insulin secretion in Rab26 KO INS-1 cells. (I) Plasma insulin levels and (J) AUC for glucose during the IPGTT. The IPGTT was performed in mice before (overnight fast) and 2 h after glucose injected intraperitoneally. (K) Blood glucose levels and (L) AUC for glucose during the IPGTT. (M) and (N) WT or KO mice were fasted for overnight before glucose levels were measured in the morning. For the ITT, blood glucose levels and AUC were measured after insulin injected intraperitoneally. (*n* = 3–9 per group; NS, not significant, ***P < 0.001, **P < 0.01, *P < 0.05, *t* tests). The numerical values that were used to generate graphs and histograms can be found in S1 Data. AUC, area under the curve; IPGTT, intraperitoneal glucose tolerance test; ITT, insulin tolerance test; KO, knockout; WT, wild type.

Rab26 was firstly identified in rat pancreas [12] and shown to regulate the maturation of exocrine granules to mediate amylase release from rat parotid acinar cells [16]. Rab26 is transcriptionally expressed in multiple tissues, and especially at higher levels in the brain (S1A Fig), and as well as in islets (S1B Fig). To study the physiological role of Rab26, we generated Rab26 gene knockout mice. The whole fragment of Rab26 gene was deleted through CRISPR/Cas9 approach using specific sgRNA targeting to the upstream of exon 1 and downstream of exon 9 of Rab26 gene (Fig 1A). The deletion of fragment was verified by DNA sequencing. The genotypes of mice were assessed by PCR approach (Fig 1B). The homozygous Rab26 gene knockout (KO) mice (Rab26^-/-^) were validated not expressing Rab26 as assessed by western blot (Fig 1C). Rab26-KO mice did not exhibit major off-target phenotypes such as lethal or infertility.

To directly assess the effect of Rab26 deficiency on insulin secretion, we isolated the islets from Rab26^-/-^ mice (as well as control mice) and measured insulin secretion in perfusion culture. Although there are no obvious morphological changes of islets (S1C Fig), the fresh islets isolated from Rab26^-/-^ mice secreted much more insulin under 16.7 mM glucose stimulation as compared to control islets (Fig 1D and 1E); in addition, depletion of Rab26 slightly promotes basal insulin secretion (S1D Fig), suggesting that absence of Rab26 promotes insulin secretion from islets to increase the circulating levels of insulin in response to glucose.

To further support our conclusion, we reduced Rab26 in mouse pancreatic insulinoma cells through CRISPR/Cas9 technique (Fig 1F and 1G). Reduction of Rab26 did not have effects on cell growth or morphological change. The stable INS-1 cell line with Rab26 reduced was used to examine insulin secretion. As shown in Fig 1H, reduction of Rab26 significantly increased insulin secretion; however, the transcripts of Ins1 (Insulin 1) and Ins2 (Insulin 2) or preproinsulin genes were not altered by Rab26 KO (S1E and S1F Fig). These results support the conclusion that deficiency of Rab26 enhances insulin secretion.

### Deficiency of Rab26 in mice improves glucose homeostasis

Insulin is the most important hormone for regulating glucose homeostasis; therefore, we examined the effects of depletion of Rab26 on glucose tolerance in mice. We first performed intraperitoneal glucose tolerance test (IPGTT) to assess the consequence of Rab26 depletion. As shown in Fig 1I and 1J, Rab26^-/-^ mice have higher insulin levels in the blood upon glucose stimulation than wild-type (WT) mice, and the glucose level in the plasma of Rab26^-/-^ mice is much lower than that of WT mice under normal feeding condition (Fig 1K and 1L). The body weight of Rab26^-/-^ mice is less than that of WT mice (S1G Fig). Intraperitoneal insulin tolerance tests (IPITTs) demonstrated that Rab26^-/-^ is more sensitive to insulin (Fig 1M and 1N), since the blood glucose levels lowered down faster in Rab26^-/-^ mice. Together with the above results, deficiency of Rab26 may improve glucose homeostasis to prevent diabetes development through enhanced insulin secretion.

### Overexpression of Rab26 restricts insulin secretion in insulinoma cells

**Fig 2:**
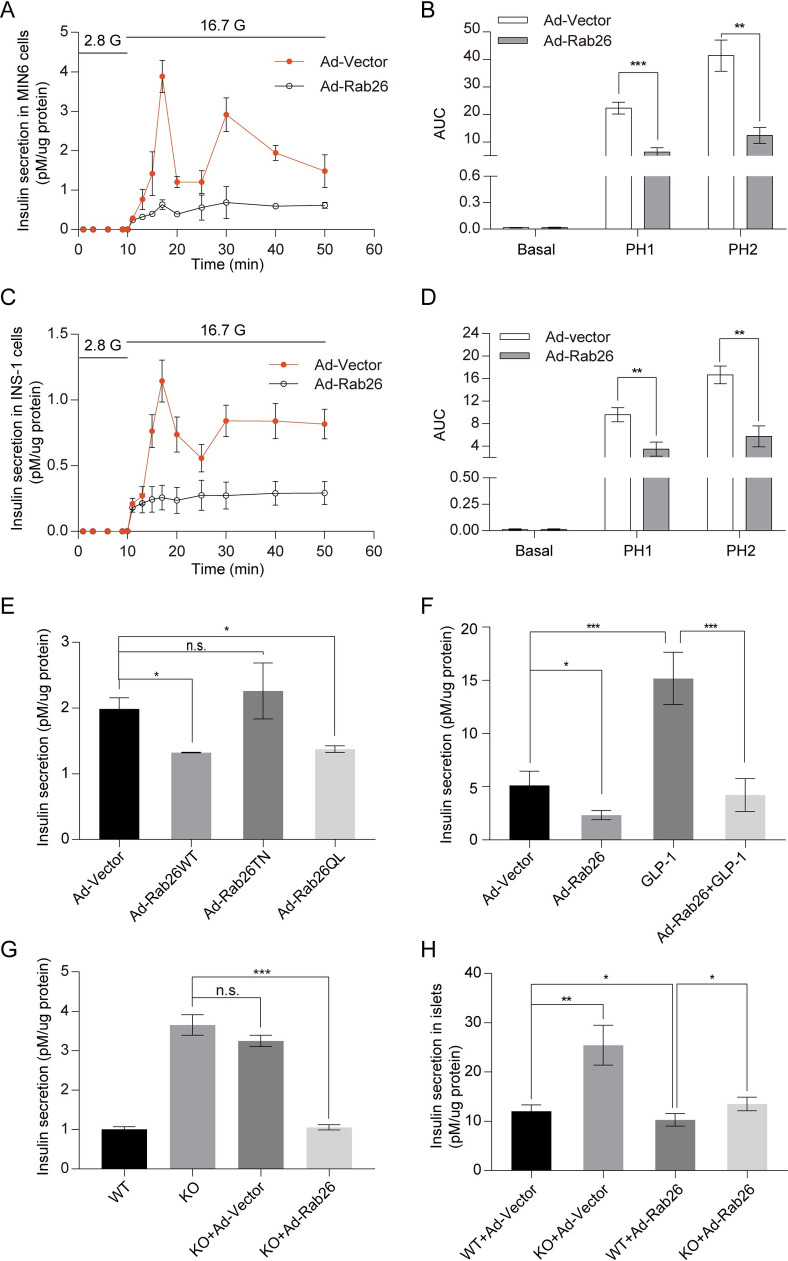
Overexpression of Rab26 restricts insulin secretion in β-cells. (A-D) MIN6 cells (A) and INS-1 cells (C) were infected with Ad-Rab26; after 40 h, cells were balanced with 2.8 mM glucose in KRBH buffer for 1 h and then stimulated with 16.7 mM glucose in KRBH buffer for at different time points. Supernatant insulin secretion was measured by ELISA, corresponding AUCs of MIN6 (B) or INS-1 (D) cells insulin release stimulated by 16.7 mM glucose stimulated. (E) INS-1 cells were infected with Ad-Rab26WT, Ad-Rab26T77N, Ad-Rab26Q123L, and Ad-vector, balanced with 2.8 mM glucose in KRBH buffer for 1 h, then stimulated with 16.7 mM glucose in KRBH buffer for 30 min. Supernatant insulin secretion was measured by ELISA. (F) INS-1 cells were transfected with Ad-vector and Ad-Rab26 alone or cocultured with 10 nM GLP-1. Supernatant insulin secretion was measured by ELISA. (G) Compared with the control, the insulin secretion of KO increased significantly, then in KO monoclonal cell line infected with Ad-Vector and Ad-Rab26, ELISA showed that Rab26 decreased insulin secretion under GSIS condition. (H) Fresh mouse islets from WT and Rab26^−/−^ mice were isolated and cultured in RPMI-1640 medium containing 10% FBS. A total of 100 islets with uniform size were selected and infected with Ad Rab26 and Ad vector, and then ELISA showed that Rab26 decreased insulin secretion under GSIS condition. (*n* = 3; NS, not significant, ***P < 0.001, **P < 0.01, *P < 0.05, *t* tests). The numerical values that were used to generate graphs and histograms can be found in S1 Data. AUC, area under the curve; GSIS, glucose-stimulated insulin secretion; KO, knockout; WT, wild-type.

As depletion of Rab26 enhanced insulin secretion, we did the converse experiment to show that overexpression of Rab26 inhibits insulin secretion. MIN6 or INS-1 were infected with the recombinant adenovirus expressing Rab26 (Ad-Rab26) (S2A and S2B Fig), and then insulin secretion was detected by ELISA assay. There are basal secretion under 2.8 mM glucose condition (S2C Fig). Overexpression of Rab26 has no major effects on the expression of Ins1, Ins2, and preproinsulin in INS-1 cells (S2D and S2E Fig). However, insulin secretion was clearly suppressed in MIN6 (Fig 2A) or INS-1 (Fig 2C) cells overexpressing Rab26 in response to glucose stimulation; corresponding AUCs of insulin release stimulated by 16.7 mM glucose were decreased in MIN6 cells (Fig 2B) and INS-1 cells (Fig 2D). In addition, the biphasic secretion pattern was disrupted in Ad-Rab26-infected MIN6 or INS-1 cells.

To examine whether the inhibitory effects of Rab26 is dependent of its guanine nucleotide binding activity, INS-1 cells were infected with Ad-Rab26, Ad-Rab26T77N (dominant negative mutant prefers binding to GDP), Ad-Rab26Q123L (constitutive active mutant with GTPase activity inhibited), or Ad-vector (S2F Fig). Cells were stimulated by glucose, then insulin secretion was detected by ELISA assay. The results demonstrated that insulin secretion was significantly suppressed by Ad-Rab26 or Ad-Rab26Q123L, but not by Ad-Rab26T66N (Fig 2E), suggesting that the inhibitory effect of Rab26 on insulin secretion is physiologically regulated by its guanine nucleotide binding activity.

GLP-1 is a glucose-dependent hormone stimulating insulin secretion [17]. We investigated the effect of Rab26 on GLP-1-stimulated insulin secretion; the results indicated that GLP-1 indeed enhanced insulin secretion. Importantly, Rab26 also suppressed GLP-1-stimulated insulin secretion (Fig 2F), indicating that Rab26 restricts the glucose-stimulated insulin secretion (GSIS) in pancreatic insulinoma cells.

As mentioned above, the depletion of Rab26 enhances insulin secretion in INS-1 cells. A rescue experiment revealed that insulin secretion was reduced upon replenishing Rab26 by infection of Ad-Rab26 in Rab26-KO INS-1 cells (Fig 2G). In addition, rescue experiments were carried out by introducing Ad-Rab26 or Ad-vector into freshly isolated islets from WT mouse or Rab26^-/-^ mice (S2G and S2H Fig). ELISA assay showed that Rab26 replenishment again inhibits insulin secretion in islets under GSIS condition (Fig 2H). Taken together, our results suggest that Rab26 serves as a negative regulator to restrict insulin secretion in pancreatic insulinoma cells and islets.

### The pathophysiological relevance of Rab26 to diabetes mellitus

**Fig 3:**
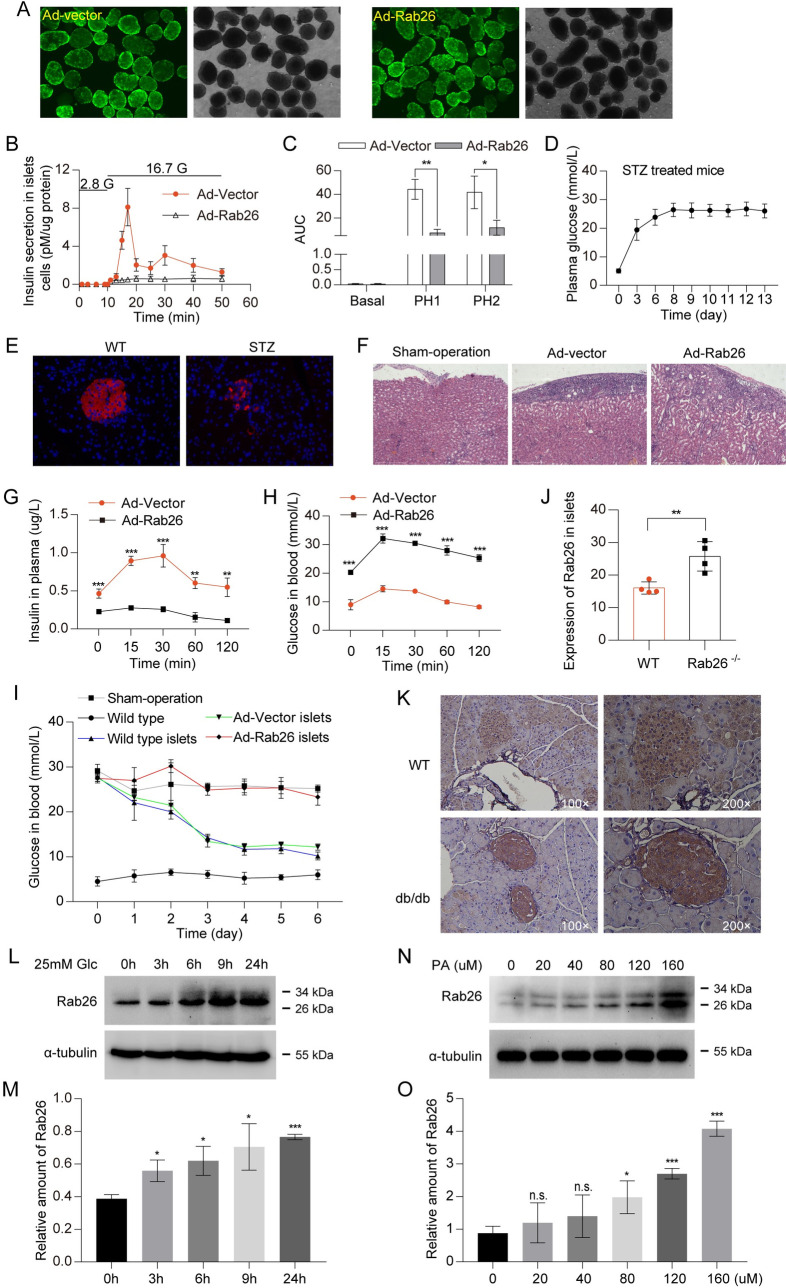
The pathophysiological relevance of Rab26 to diabetes mellitus. (A) Fresh mouse islets were isolated and cultured in 1640 medium containing 10% FBS. A total of 100 islets of uniform size were selected and infected with Ad-Rab26 and Ad-vector, (B) then insulin secretion of mouse islets assessed by ELISA at the indicated time. (C) AUC of B were calculated. (D) After intraperitoneal injection of streptozotocin, the blood glucose of 20–22 g male C57BL/6J mice increased. (E) Normal mouse islets (left panel) and injected streptozotocin mice islets (right panel), insulin antibody immunofluorescence staining showed that the right islets were destroyed, indicating that the model of type 1 diabetic mice was successful. (F) HE staining of mice renal capsule performed with islets transplantation. (G) The islets infected by Ad-Rab26 were transplanted into type 1 diabetic mice. In IPGTT experiment, the level of plasma insulin in mice was detected at 0 min, 15 min, 30 min, 60 min, and 120 min time points after transplantation for 5 d. (H) Blood glucose levels were measured from tail vein. (I) Compared with normal mice, the blood glucose of type 1 diabetic mice with islet transplantation decreased, but the blood glucose of mice infected with Ad-Rab26 could not be reduced within 6 d after transplantation of islets. (J and K) Representative immunohistochemical images indicating Rab26 protein expression in WT and *db/db* mice pancreas. J is the quantitative data from K. The images were performed with 4 mice pancreas in each of the 2 groups. (L) Western blot showed that Rab26 protein expression increased after 25 mM glucose stimulation for different time. (M) Quantitative analysis of the results of J from 3 independent experiments. (N) To test the dose-dependent effect of PA on INS-1 cells, cells were treated with 0, 20, 40, 80, 120, and 160 μM PA for 48 h. Western blot showed that Rab26 protein expression increased after PA stimulation for different concentration. (O) Quantitative analysis of the results of L from 3 independent experiments. (*n* = 3–5; NS, not significant, ***P < 0.001, **P < 0.01, *P < 0.05, *t* tests). The numerical values that were used to generate graphs and histograms can be found in S1 Data. AUC, area under the curve; HE, hematoxylin–eosin; IPGTT, intraperitoneal glucose tolerance test; PA, palmitic acid; WT, wild-type.

To further define the role of Rab26 in insulin secretion, freshly isolated mouse islets were effectively infected with Ad-Rab26 (Fig 3A). The islets was stimulated by 16.7 mM glucose in KRBH buffer, the secreted insulin was detected by ELISA assay; as shown in Fig 3B and 3C, overexpression of Rab26 significantly inhibited insulin secretion from islets at different detection time points compared with islets transduced with vector control, suggesting that overexpression of Rab26 suppresses insulin secretion not only in insulinoma cells, but also in freshly isolated islets, which is close to the in vivo physiological condition.

Next, we examined whether Rab26 affected the function of islets in vivo. The mouse model of type 1 diabetes was established by intraperitoneal injection of streptozotocin to destroy β cells in the islets (Fig 3D). Immunofluorescence staining with insulin antibody showed that injection of streptozotocin destroyed the islets accompanied with high blood glucose levels, indicating that the model of type 1 diabetic mice was generated (Fig 3E). Islets infected with Ad-Rab26 or vector were transplanted back to type 1 diabetic mice beneath the renal capsule (Fig 3F). The transplanted mice were intraperitoneally injected with glucose, then the plasma insulin and blood glucose were monitored. As expected, the plasma insulin levels of type 1 diabetic mice transplanted with islets overexpressing Rab26 were much lower than that of control mice (Fig 3G). Meanwhile, the blood glucose levels of mice transplanted with islets overexpressing Rab26 decreased more slowly and kept at high level for longer period of time (continuously monitored for 6 d Fig 3H and 3I). The mice transplanted with Ad-Rab26 islets exhibits hyperglycemia with glucose levels up to 25 mM, while the glucose levels of control mice were restored to normal (Fig 3H and 3I). The results suggest that overexpression of Rab26 not only restricted insulin secretion but also suppressed the rescuing function of isolated islets upon transplantation into type I diabetic experimental model.

Since Rab26 is involved in insulin secretion, the expression of Rab26 may be related to diabetes mellitus. Immunohistochemistry analysis found that the staining signals of Rab26 in islet of db/db mice are stronger than that of WT mice (Fig 3J and 3K), indicating high levels of Rab26 protein in islets of db/db mice. Furthermore, Rab26 protein level was elevated under high glucose (Fig 3L and 3M) or high palmitic acid conditions (Fig 3N and 3O). These results suggest that the expression of Rab26 is closely related to diabetic pathophysiological conditions.

### Rab26 associates with insulin granules and regulates the distribution of insulin granules

**Fig 4:**
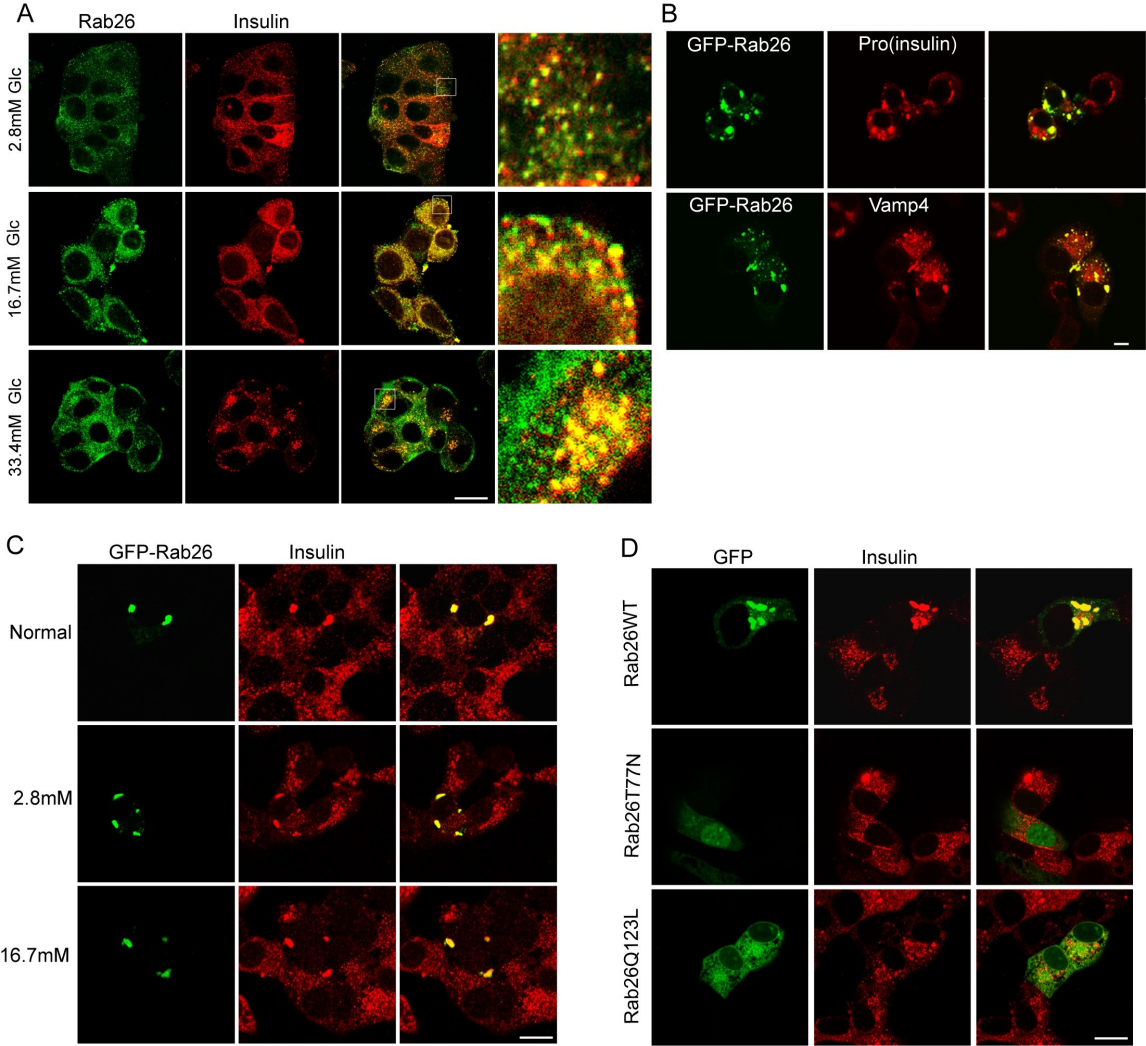
Rab26 induces clustering and enlargement of insulin particles. (A) INS-1 cell were double-labeled with anti-Rab26 and anti-insulin under 2.8 mM, 16.7 mM, or 33.4 mM glucose condition. (B) MIN6 cells transfected with GFP-Rab26 were cultured in KRB buffer containing 16.7 mM glucose for 40 min, and immunofluorescence microscopy revealed that GFP-Rab26 was expressed in puncta, which colocalized with pro(insulin) and Vamp4. (C) In MIN6 cells transiently transfected plasmids, insulin were similarly dispersed throughout the cytosol and displayed a similar extent of colocalization with Rab26WT. (D) MIN6 cells were transfected with GFP-Rab26WT, Rab26T77N, or Rab26Q123L and labeled with insulin. Bar = 20 μm.

To investigate how Rab26 regulates insulin secretion, we examined the subcellular location of Rab26 and the effects of its overexpression on the distribution of insulin granules in insulinoma cells. Rab26 was shown to be associated with the endocytic compartments [18]. Immunostaining results for the endogenous Rab26 using Rab26 antibody revealed that over 50% Rab26-containing vesicles colocalize with insulin granules under different glucose conditions in INS-1 cells (Fig 4A). In addition, immunofluorescence microscopy revealed that GFP-Rab26 was present in puncta, which colocalized with pro (insulin) and Vamp4 in MIN6 cells (Fig 4B). These results suggest that Rab26 physiologically associates with the secretory vesicles and insulin granules and is supposed to be a regulator for insulin secretion. Further examinations demonstrated that overexpression of Rab26 resulted in insulin granules clustering in normal media, KRB buffer containing 2.8 mM glucose or 16.7 mM glucose in MIN6 cells (Fig 4C). Under normal conditions, overexpression of Rab26WT or Rab26Q123L mutant induced insulin granules clustering, while the inactivated mutant Rab26T77N was distributed in the cytosol and did not induce insulin granule granules clustering (Fig 4D). Consistent with the results that Rab26WT and Rab26Q123L but not Rab26T77N inhibit insulin secretion, this clustering distribution of insulin granules induced by Rab26 may inhibit glucose stimulation triggered exocytosis of insulin granules and consequently restrict insulin secretion.

### Rab26 interacts with synaptotagmin-1

**Fig 5:**
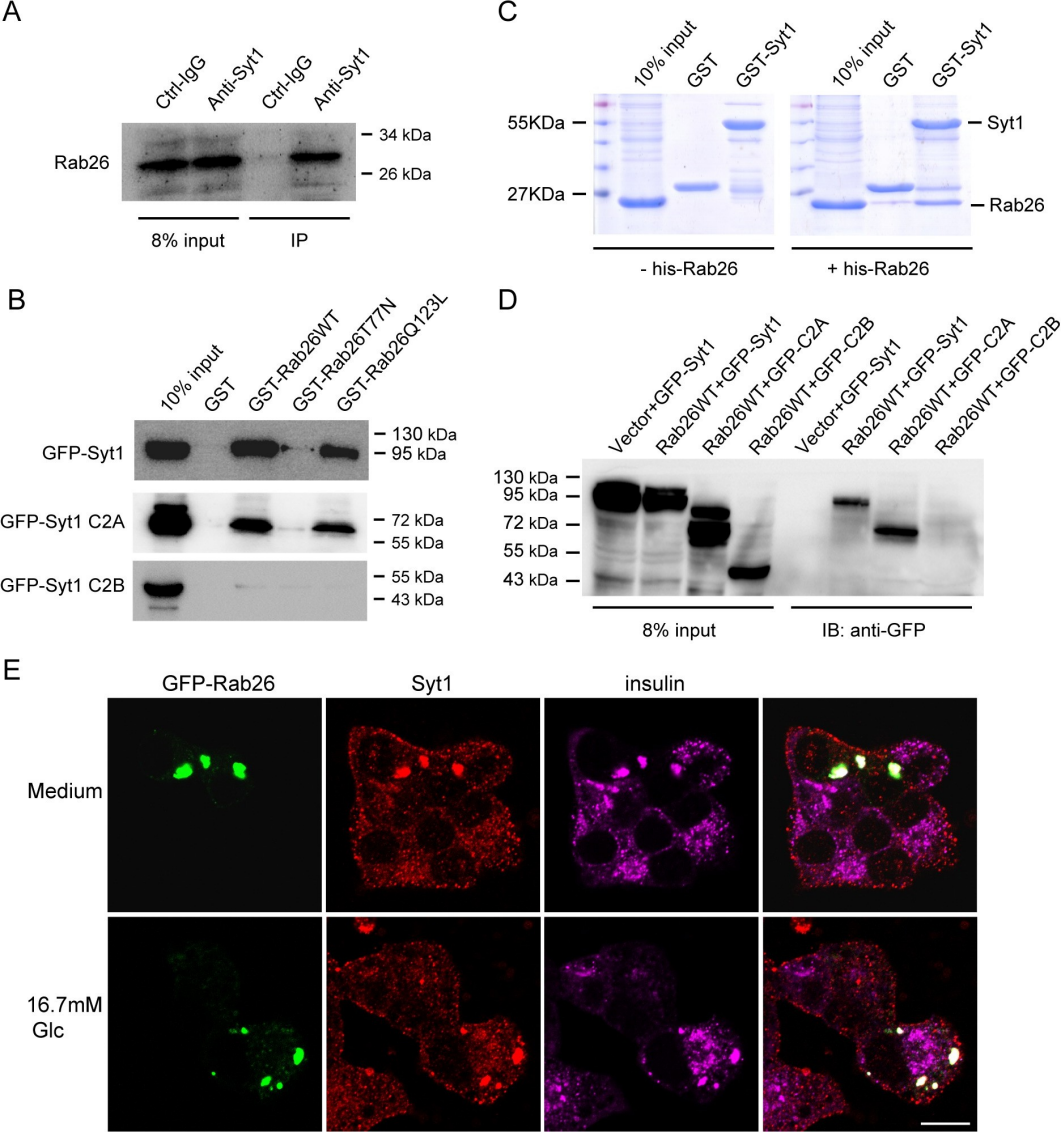
Rab26 directly interacts with Syt1. (A) co-IP experiments using Syt1 antibody to precipitate the endogenous Rab26 in INS-1 cell lysates. (B) GST, GST-Rab26WT, GST-Rab26T77N, and GST-Rab26Q123L were mixed with the 293T cell lysates containing GFP-Syt1, Syt1-C2A, or C2B, respectively. Western blotting analysis was performed with GFP antibody. (C) In vitro binding assay showed His-Rab26 can directly bind to GST-Syt1. (D) GFP-Syt1 and GST-Vector, GFP-Syt1 and GST-Rab26, GFP-Syt1-C2A and GST-Rab26, GFP-Syt1-C2B and GST-Rab26, cell lysate was mixed, and after extensive washing. After a large number of washing, western blotting analysis was performed with GFP antibody. (E) MIN6 cells transfected with GFP-Rab26 were cultured in normal medium or in KRB buffer containing 16.7 mM glucose for 40 min and then immunostained with antibody against Syt1 and insulin, showing that Rab26 induces Syt1 and insulin granules clustering. Bar = 20 μm.

To uncover the molecular mechanism for Rab26 in regulating insulin secretion, we searched for its interacting proteins. Syt1 along with several other proteins were identified as potential interacting partners of Rab26 from rat brain tissue lysate in large scale GST-pulldown experiments (S3A Fig). Syt1 is an important calcium sensor to regulate neurotransmitter release in neuron and insulin secretion in pancreatic β-cells [19,20]. To confirm the interaction between Rab26 and Syt1, we performed co-IP experiments using Syt1 antibody to precipitate the endogenous Rab26 in INS-1 cell lysates (Fig 5A); the results revealed that Rab26 interacts with Syt1 in INS-1 cells. The interaction of Rab26 with Syt1 was validated by analytic GST-pulldown assay. The interaction between Rab26 and Syt1 depends on Rab26’s nucleotide binding activity, as the interaction between GDP-bound mutant Rab26T77N and Syt1 was dramatically reduced (Fig 5B). In vitro binding assay using prokaryotic-expressed GST-Syt1 and His-Rab26 showed that Rab26 can directly bind to Syt1 (Fig 5C). In addition, GST-pulldown experiments using GST-Rab26 revealed that Rab26 only interacts with Syt1-C2A domain, but not C2B domain in cell lysates derived from cells transfected with GFP-Syt1, GFP-Syt1-C2A, or GFP-Syt1-C2B (Fig 5D). Again, Rab26 binds directly Syt1 through interacting with the C2A domain in a manner dependent on its binding with GTP (Fig 5B). Besides Syt1, other members of Synaptotagmin such as Syt4 and Syt7 may regulate insulin secretion as well [21–23]. However, Rab26 did not interact with Syt4 or Syt7 (S3B and S3C Fig), suggesting that Rab26 specifically interacts with Syt1.

### The interaction between Rab26 and Syt1 is essential to its inhibitory activity for insulin secretion

To investigate whether Rab26 restricts insulin secretion through interaction with Syt1, we first examined whether Rab26 associates with Syt1 at insulin granules. Immunofluorescence microscopy revealed that Rab26 is colocalized with Syt1 and insulin (Fig 5E). GFP-Rab26 also induced Syt1-containing insulin granules clustering in MIN6 cells under normal culture condition or glucose stimulation. These results imply that Rab26 can associate with insulin granules by binding to Syt1 and consequently regulates exocytosis of insulin granules.

**Fig 6:**
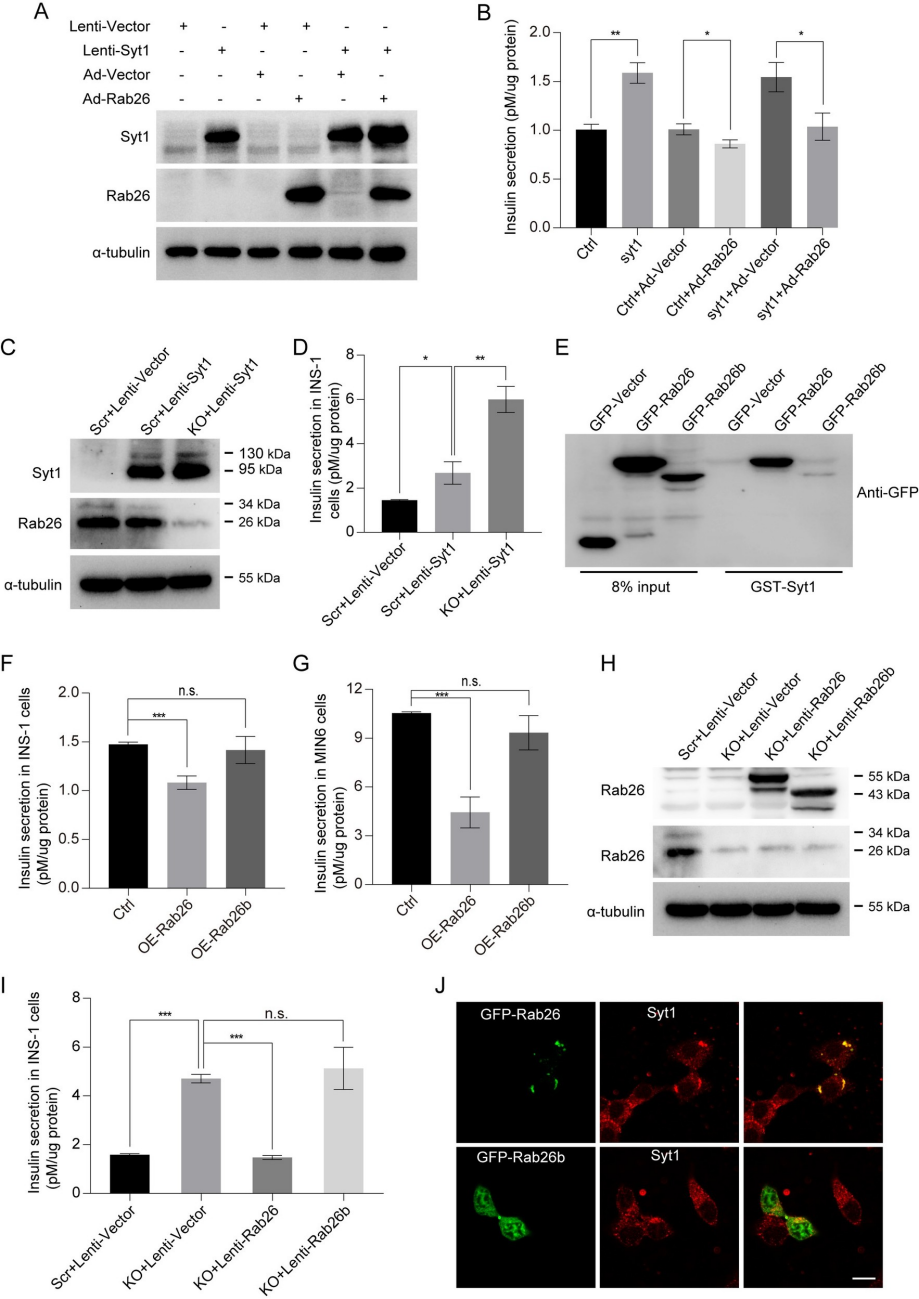
The interaction between Rab26 and Syt1 is essential to its inhibitory activity for insulin secretion. (A) and (B) INS-1 cells were infected with lenti-syt1, then infected with Ad-Rab26, the corresponding cells were stimulated for insulin secretion in KRBH buffer under GSIS condition, insulin secretion were assessed by ELISA, and western blot was used to detect Syt1, Rab26, and α-tubulin. (C) and (D) INS-1 cells were infected with Scr+lenti-Vector, Scr+lenti-Syt1, and KO+lenti-Syt1, the corresponding cells were stimulated for insulin secretion in KRBH buffer under GSIS condition, insulin secretion were assessed by ELISA, and western blot was used to detect Syt1, Rab26, and α-tubulin. (E) 293t cells were transfected GFP-Vector, GFP-Rab26, or GFP-Rab26b, and the derived cells lysates were incubated overnight with GST-syt1 beads at 4°C through GST-pulldown experiment. (F) and (G) INS-1 cells and MIN6 cells were infected with the Ad-vector, Ad-Rab26, or Ad-Rab26b, after 48 h, and balanced in KRBH buffer with 2.8 mM glucose for 1 h, then stimulated with glucose(16.7 mM) for 30 min. Insulin secretion was assessed by ELISA. (H) and (I) INS-1 cells were infected with Scr+lenti-Vector, KO+lenti-Vector, KO+lenti-Rab26, and KO+lenti-Rab26b, the corresponding cells were stimulated for insulin secretion in KRBH buffer under GSIS condition, insulin secretion were assessed by ELISA, and western blot was used to detect Rab26 and α-tubulin. (J) MIN6 cells transfected with GFP-Rab26 or GFP-Rab26b were cultured in normal medium and then immunostained with antibody against Syt1. (*n* = 3–5; NS, not significant, ***P < 0.001, **P < 0.01, *P < 0.05, *t* tests). The numerical values that were used to generate graphs and histograms can be found in S1 Data. GSIS, glucose-stimulated insulin secretion; KO, knockout; Syt1, Synaptotagmin-1.

Overexpression of Syt1 promotes insulin secretion [23]. We prepared lentivirus-mediated overexpression of Syt1 in INS-1 cells; the cells then were infected with Ad-Rab26 (Fig 6A). Insulin secretion was monitored by ELISA assay. Indeed, overexpression of Syt1 enhanced insulin secretion in INS-1 cells (Fig 6B). However, insulin secretion enhanced by Syt1 was significantly suppressed when Ad-Rab26 was coexpressed with Syt1 (Fig 6B). Additionally, overexpression of Syt1 greatly enhances insulin secretion in Rab26-KO INS-1 cells (Fig 6C and 6D); these results suggest that Rab26 inhibits insulin secretion through interaction with Syt1.

By examining Genbank, we found that Rab26 gene encodes another shorter isoform (accession no. NM_001308053.1) referred to as Rab26b [24]. Rab26b protein lacks N-terminal 66 amino acids. Interestingly, Rab26b was not able to interact with Syt1 in GST-pulldown experiments (Fig 6E), which was verified by using GST-Rab26 or GST-Rab26b to bind GFP-Syt1 (S3D Fig). Rab26b had no significant inhibitory effects on glucose-stimulated insulin secretion in both INS-1 and MIN6 cells (Fig 6F and 6G). As expected, Rab26b has no inhibitory effects on Rab26-KO-resulted insulin secretion enhancement (Fig 6H and 6I). In addition, Rab26b was not recruited to Syt1 containing insulin granules and was distributed primarily in the cytosol, not inducing granules clustering either (Fig 6J). These results suggest that N-terminal extension of Rab26 as compared to Rab26b is necessary for interaction with Syt1, clustering insulin granules or restricting insulin secretion. Taken together, Rab26 restricts insulin secretion via interacting directly with C2A region of Syt1 in a manner that is dependent on the N-terminal region and GTP-bound status of Rab26.

### Rab26 influences the interaction between Syt1 and SNARE complex

**Fig 7:**
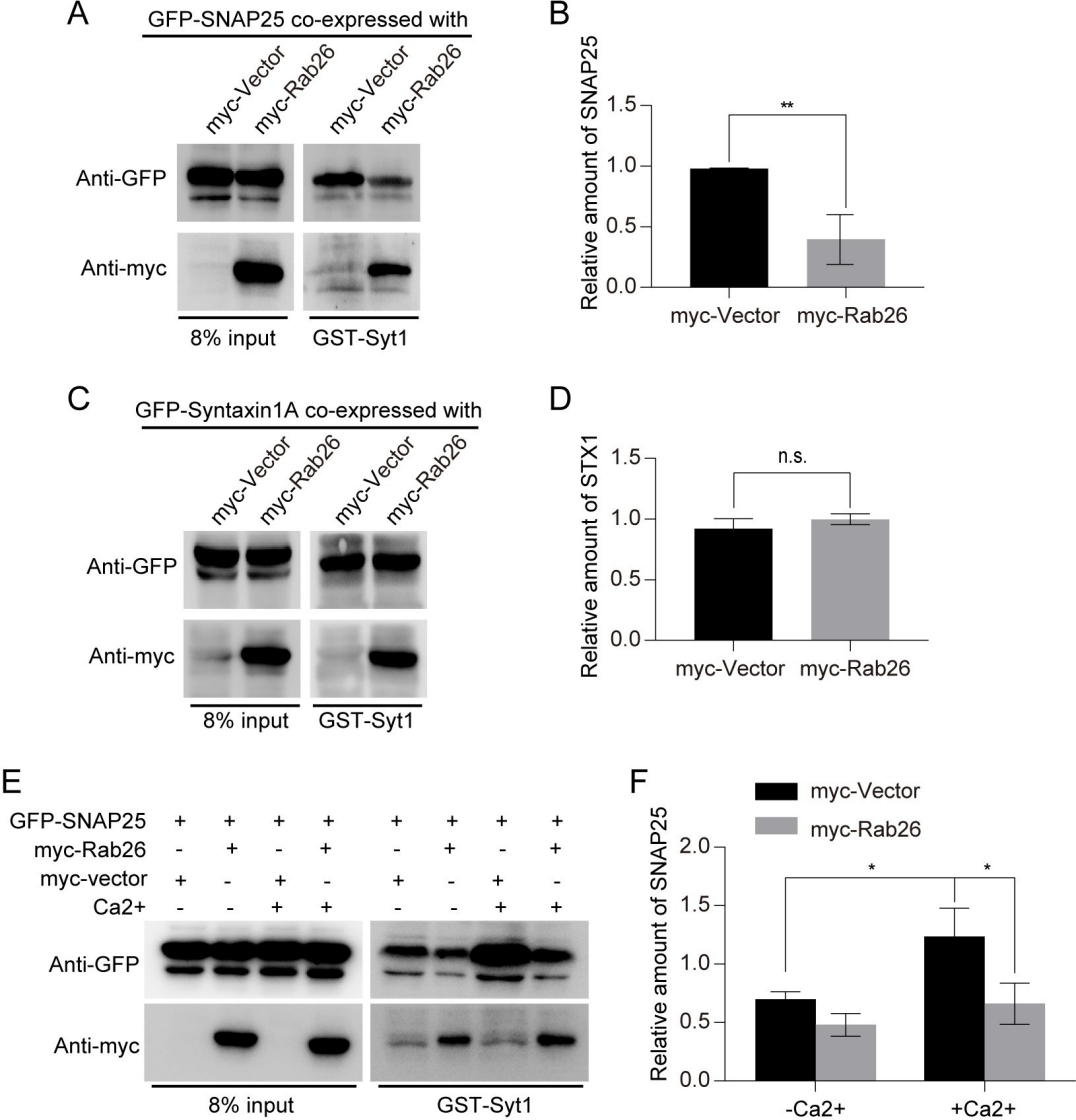
Rab26 influences the interaction between Syt1 and SNARE complex. (A) 293t cells were cotransfected myc-Vector and GFP-SNAP25 as control group, myc-Rab26 and GFP-SNAP25 as experimental group, incubated overnight with GST-Syt1 beads at 4°C through GST-pulldown experiment, washed by lysis buffer, and analyzed by western blotting with GFP antibody. (B) Quantitative analysis of the results of A from 3 independent experiments. (C) 293t cells were transfected myc-Vector and GFP-Syntaxin 1A as control group, myc-Rab26 and GFP-Syntaxin 1A as experimental group, incubated with GST-Syt1 beads, and analyzed by western blotting with GFP antibody. (D) Quantitative analysis of the results of C from 3 independent experiments. (A), (E) Cells were incubated for 5 min at 37°C in the presence of Ca^2+^ (10 μM CaCl2) or absence of Ca^2+^, indicating that Rab26 reduces the interaction between Syt1 and SNAP25. (F) Quantitative analysis of the results of E from 3 independent experiments. (*n* = 3; NS, not significant, ***P < 0.001, **P < 0.01, *P < 0.05, *t* tests). The numerical values that were used to generate graphs and histograms can be found in S1 Data.

Since Syt1 facilitates secretory vesicles docking to and fusion with plasma membrane through interaction with SNARE complex [25], we next examined whether Rab26 influences the interaction between Syt1 and SNARE complex. 293t cells were cotransfected with myc-Rab26 and GFP-SNAP25 or GFP-Syntaxin-1; the cell lysates were subjected for GST-pulldown using GST-Syt1. As shown in Fig 7A and 7B, the amount of SNAP25 protein bound to GST-Syt1 was significantly reduced in cells expressing Rab26 compared with control vector, though overexpression of Rab26 does not alter the protein levels of Syt1, Syntaxin1, and SNAP25 (S4A Fig). However, expression of Rab26 had no effects on Syt1 binding to Syntaxin-1 (Fig 7C and 7D). Ca^2+^ stimulation promoted Syt1 binding to SNAP25, which was inhibited by Rab26 expression (Fig 7E and 7F). These results suggest that interaction of Rab26 with Syt1 inhibits Syt1 binding to SNAP25. Rab26 thus may restrict insulin secretion via sequestering Syt1 from interacting with SNAP-25.

As Syt1 can bind to phospholipids, we investigated whether Rab26 interaction with Syt1 influences Syt1 binding to phospholipids. In vitro overlay assay revealed that Rab26 did not affect the interaction of Syt1 with PI(4,5)P_2_ and PS (S4B Fig). Therefore, Rab26 inhibits insulin secretory granules exocytosis probably by interfering Syt1 interaction with SNARE complex and consequently inhibits insulin secretory granules fusion with the plasma membrane.

### Rab26 inhibits exocytosis of newcomer insulin granules

Rab26 inhibits SNAP25 binding to Syt1, suggesting that Rab26 may influence Syt1 interaction with SNARE complex and consequently insulin secretory granules (ISGs) fusion with the plasma membrane. To examine this hypothesis, MIN6 cells were cotransfected with GFP-Rab26 and DsRed-Insulin and then analyzed for the ISGs dynamics by employing time-lapse total internal reflection fluorescence microscopy (TIRFM).

**Fig 8:**
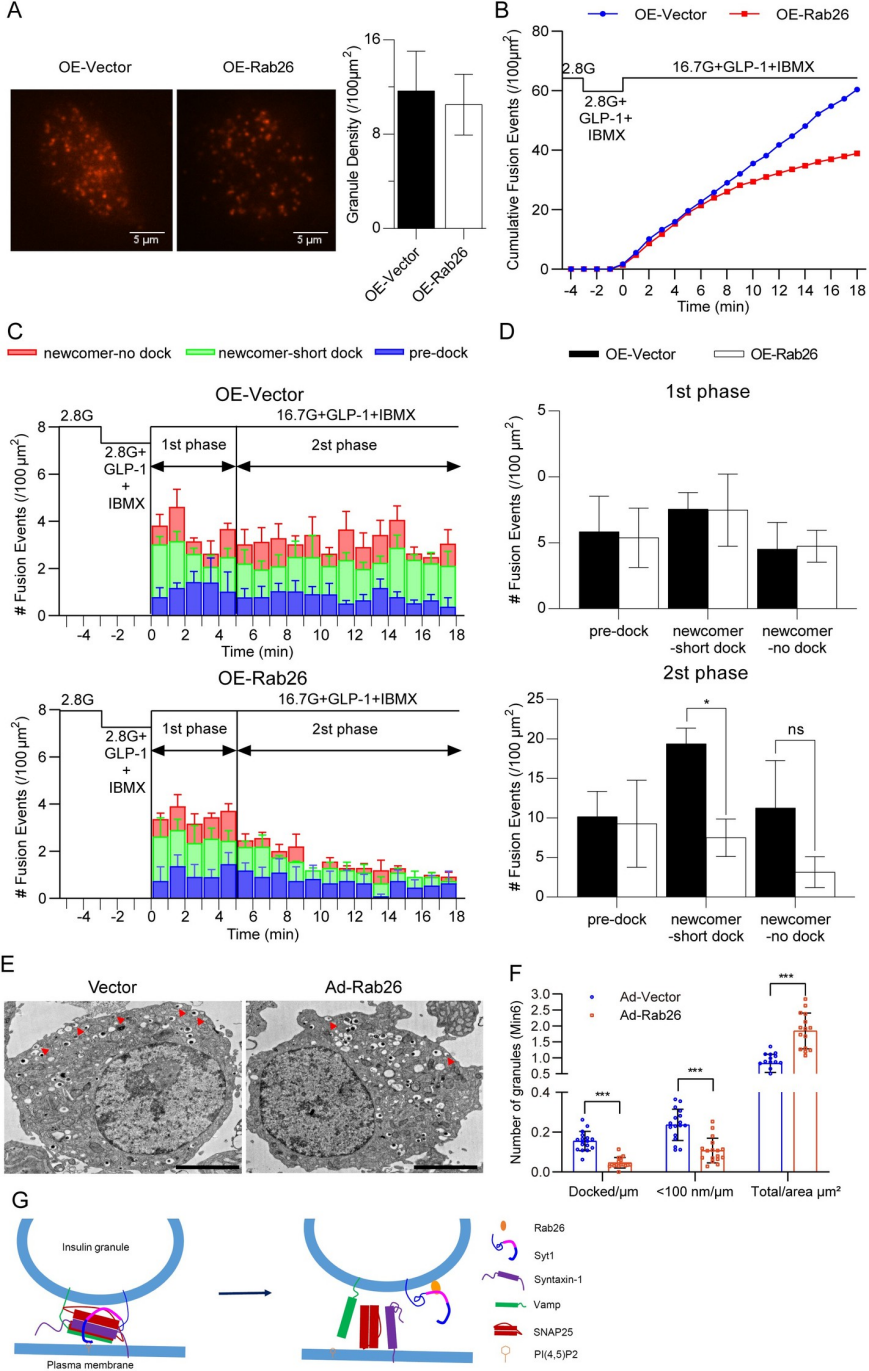
Rab26 inhibits exocytosis of newcomer insulin granules. (A) TIRF images of docked insulin granules in vector-transfected (OE-Vector) or Rab26-transfected (OE-Rab26) MIN6 cells; there is no significant difference in the averaged SG densities before stimulation. Data were collected from 3 experiments, and 3 cells were analyzed in each experiment, shown as means ± SEMs. (B) Normalized cumulative fusion events of insulin granules per 100 μm^2^ from OE-Vector and OE-Rab26 MIN6 cells. (C) The insulin SGs exocytosis dynamics evoked by 16.7 mM glucose from OE-Vector versus OE-Rab26 MIN6 cells. Data were obtained from 3 experiments, and 3 cells were analyzed in each experiments, shown as means ± SEMs. Blue, green, and red bars indicate the insulin granules pre-dock, newcomer-short dock, and newcomer-no dock, respectively. (D) Summary of the fusion events from 3 modes: pre-dock, newcomer-short dock, and newcomer-no dock in the first phase and second phase after 16.7 mM glucose stimulation. (E) The representative TEM images of MIN6 cell infected with Ad-vector or Ad-Rab26; the arrow head indicated the granules docked on plasma membrane. (F) Quantitative analysis data showing the number of insulin granules docked on plasma membrane, <100 nm from plasma membrane and the total insulin granules. (G) A model proposes Rab26 interacting with Syt1 to sequester it from promoting the formation of SNARE complex involved in the docking and fusion of insulin granules. The numerical values that were used to generate graphs and histograms can be found in S1 Data. SG, secretory granule; Syt1, Synaptotagmin-1; TIRF, total internal reflection fluorescence.

The exocytotic events were, however, not uniform but could be categorized into 3 distinct modes of exocytosis. Pre-dock SGs that were visible before stimulation, no-dock newcomers SGs that fused without remaining at the plasma membrane for ≤200 milliseconds (interval of 1 frame), and short-dock newcomers SGs that appeared during stimulation and stably remained for >200 milliseconds before fusion occurs. The number of pre-docked SGs was counted and averaged at the first 2 min prior to stimulation. At unstimulated state, the average number of pre-docked ISGs in Rab26-transfected cells is similar to that of vector-transfected cells (Fig 8A); when stimulated with 16.7 mM glucose (containing GLP-1 and IBMX), the accumulated fusion events in Rab26-transfected cells were significantly less than that in vector-transfected cells (Fig 8B). Single SG fusion dynamic analysis revealed that no fusion events were observed before stimulation. Both the newcomer SGs no-dock and newcomer SGs short-dock were decreased in Rab26-transfected cells compared to that in vector-transfected cells, and not displaying two-phase secretion pattern in Rab26-transfected cells (Fig 8C), which is consistent with the results shown in Fig 2A and 2B. The fusion events summarized from newcomer no-dock SGs and newcomer SGs short-dock demonstrated that Rab26 significantly inhibits the newcomer SGs fusion with the plasma membrane (Fig 8D). MIN6 cells expressing Ad-Rab26 were processed for transmission electronic microscopy analysis; it is observed that overexpression of Rab26 reduces the number of docked granules on plasma membrane and slightly increases the total number of granules (Fig 8E and 8F). Taken together, Rab26 interaction with Syt1 may interfere Syt1 interaction with SNARE complex and consequently inhibit ISGs exocytosis, resulting in the inhibition of insulin secretion.

## Discussion

Rab26 is implicated in multiple vesicular trafficking events, such as autophagy, exocrine granule release, synaptic vesicle trafficking, and mitochondrial distribution [14,26,27]. Our earlier study showing that Rab7-interacting lysosomal protein (RILP) is involved in insulin secretion prompted us to investigate the role of Rab26 in insulin secretion. In this study, we generated Rab26 gene KO mice and found that deficiency of Rab26 enhances insulin secretion and improves glucose homeostasis in diabetic mice, suggesting its role in restricting insulin secretion. Consistently, Rab26 protein level in islets of diabetic mice is elevated, as well as under high glucose or fatty acid conditions.

Rab26 is a transcriptional target of MIST1 that regulates the formation of exocrine secretory granules in human gastric cancer cell [28]. Our results showed that depletion of Rab26 promotes insulin secretion in mice or pancreatic insulinoma cells, while overexpression of Rab26 inhibits insulin secretion in both beta cell lines and freshly isolated islets. These findings suggest that Rab26 exerts a different function in regulating secretory pathway in pancreatic insulinoma cells. Coincidently, RILP also restricts insulin secretion through mediating proinsulin degradation [18]. By contrary, Rab3 and Rab27 positively regulate insulin secretion [10,29], indicating that insulin secretion is tightly regulated by diverse mechanisms to maintain its homeostasis.

More than 10 members of Synaptotagmin family were identified, and several of them have been established to regulate Ca^2+^ triggered insulin granules exocytosis [22,23,30,31]. Syt1 is the most important Ca^2+^ sensor in regulating insulin secretion in pancreatic β-cells [20,32]. Syt1 has 2 regulatory domains, C2A and C2B, which interact with SNARE (soluble NSF-associated protein receptor) SNAP25/Syntaxin1/Vamp, as well as phosphatidylinositol-(4,5) bisphosphate on the plasma membrane, thus mediating Ca ^2+^ triggered secretory vesicles docking and fusion with plasma membrane [33–37]. Mechanistically, Rab26 directly binds to the C2A domain of Syt1. We also demonstrated that Rab26-Syt1 interaction is essential to Rab26’s inhibitory activity on insulin secretion, as an isoform Rab26b not interacting with Syt1 does not inhibit insulin secretion. Although Syt1 may interact with SNARE SNAP25 and Syntaxin1, in our study, overexpression of Rab26 only specifically interferes the interaction between Syt1 and SNAP25, suggesting that SNAP25 and Syntaxin-1 may bind to the different region or motifs of Syt1, which deserves further examination.

Syt1 interaction with SNARE may help SNARE complex clamping or zippering [38,39]. In addition, Syt1 binds to PI(4,5)P_2_ to help insulin granule docking [40,41]. Nevertheless, the precise mechanisms for Syt1 regulating exocytosis remains to be elucidated. C2B domain of Syt1 seems more flexible to interact with accessary partners; however, there is some cooperation between C2A and C2B, and they can influence each other [42,43]. Therefore, Rab26 interaction with C2A domain may influence the function of C2B. Although we presented data indicating that the interaction between Rab26 and Syt1 influences SNARE complex formation, more precise details need to be worked in future studies. Therefore, Rab26 interacting with C2A domain of Syt1 will sequester it from promoting the formation of SNARE complex involved in the docking and fusion of insulin granules (Fig 8G). As Syt1 also interacts with Rab3 and Rab27 (S3E Fig), Rab26 may compete with Rab3 or Rab27 to interact with Syt1, suggesting another mechanism for Rab26 regulating SNARE function.

Rab26’s interaction with Syt1, its role in clustering of insulin granules, and its inhibition on insulin secretion are all dependent on its GTP-bound state as the WT, GTP-bound mutant but not GDP-restricted mutant displayed these effects. Collectively, these results suggest that Rab26 restricts insulin secretion via sequestering Syt1 on insulin granule to suppress the docking and fusion. This conclusion raises the interesting possibilities that proteins regulating GTP cycle of Rab26 such as its GEFs and GAPs are likely involved in regulating insulin secretion. These candidate GEFs and GAPs may receive other physiological inputs so that insulin secretion is precisely regulated, although their identities remain to be identified.

To maintain insulin balance, excess subcellular insulin may be channeled to degradation pathway [44,45]. Autophagy is the major pathway for insulin granules degradation [46]. Particularly in β cells, other machineries are engaged in sustaining insulin homeostasis; for example, the ISGs can be digested via lysosomal degradation (crinophagy) [47] or starvation-induced nascent granule degradation (SINGD) instead of autophagy [48]. Golgi membrane-associated degradation (GOMED) pathway is the compensatory pathway for the granules degradation when crinophagy and SINGD are suppressed [49]. BIG3 is a member of Arf-GTP exchange factor, which negatively modulates insulin granule biogenesis and insulin secretion [50]. RILP mediates lysosomal degradation of proinsulin to restrict insulin secretion [18]. Rab26 restricts insulin granules exocytosis, resulting in the accumulated/clustered insulin granules, since Rab26 also mediates autophagic pathway [13,14]. Our data also revealed that Rab26 slightly elevates the levels of LC3II (S5 Fig). Therefore, the Rab26-resulted accumulated/clustered granules may potentially be directed to degradation through the abovementioned degradation pathway to maintain insulin homeostasis.

In summary, our results uncover a novel function of Rab26, which negatively regulates insulin secretion. Mechanistically, Rab26 interacts with Syt1 to interfere SNAP25-Syt1 interaction, consequently influencing insulin granule docking and fusion with plasma membrane.

## Materials and methods

### Ethics statement

All animal experiments were approved by Institutional Animal Ethics Committee of Xiamen University, China, with experimental animal ethics approval No.XMULAC20180125.

### Generation of Rab26 gene knockout mice

C57BL/6J mouse strain was used for generating gene KO model. Rab26 gene KO mice were generated using CRISPR/Cas9 approach. sgRNA-1 (5-GACCCGAACCCGTCCCGCAGCGG-3) and sgRNA-2 (5-TAGACTCGGGCAATTCTCAAAGG-3) targeting to the flanking region 5′ of exon 1 and 3′ of last exon of Rab26 gene, respectively, were used to delete the whole genomic region encoding Rab26 in mouse embryonic stem cells. The genotypes of mice were verified by tail DNA PCR using specific forward primer 1 (5-GACAACTGGAGCCCTTTGAG-3), reverse primer 2 (5-GGCCTTGCAGTAGATGGAGT-3), and mutant reverser primer 3 (5-GACGGTATCAGCGCATGTGT-3) and western blot assay using Rab26 antibody.

### Cell culture and transfection

MIN6, INS-1, 293t, and 293A cell lines were from ATCC (American Type Culture Collection). MIN6 cells were maintained in Dulbecco’s Modified Eagle Medium (DMEM, Invitrogen, cat. No.12800017) supplemented with 10% FBS and 50 μM β-Mercaptoethanol in 5% CO2 incubator at 37°C. INS-1 cells were maintained in RPMI-1640 supplemented with 10% FBS, 10 mM Hepes, 1 mM PyrNa, and 50 μM β-Mercaptoethanol in 5% CO_2_ incubator at 37°C. Cells were transfected by using Lipofactamine2000 reagents (Invitrogen) according to the manufacturer’s protocol.

### Antibodies and reagents

Rab26 rabbit monoclonal antibody (mAb) was purchased from Proteintech (cat.14284-1AP). Rab26 mouse mAb was purchased from Synaptic Systems (cat. no.269011). Rabbit polyclonal antibody against insulin (cat. no. 4590 s) was from Cell Signaling Technology. Proinsulin mAb was obtained from HyTest (cat. no. CCI-17). mAb against α-tubulin (cat.66031-1-Ig) and GFP (cat.66002-1-Ig) were from Proteintech (Wuhan, China). mAb against syt1 was purchased from Synaptic Systems (cat. no.105011). mAb against Myc (9E10) was obtained from ATCC (Manassas, VA, USA). Horseradish peroxidase (HRP)-conjugated secondary antibodies, Cy5-conjugated, Texas red-conjugated were from Jackson Immuno Research (cat. 111-035-003, 115-025-003, 111-175-144, 115-295-003, West Grove, PA, USA). GLP-1 (Glucagon-like peptide-1) and IBMX (Isobutyl-1-methylxanthine) was purchased from MCE (MedChemExpress). Collagenase V, STZ (Streptozotocin), and PA (Palmitic Acid) were purchased from Sigma-Aldrich.

### Expression plasmids and virus-mediated gene expression

GFP-Rab26WT, GFP-Rab26Q123L, GFP-Rab26T77N, myc-Rab26, GST-Rab26WT, GST-Rab26Q123L, GST-Rab26T77N, GFP-Rab26b, and GST-Rab26b were described previously [18]. Syt1 cDNA was retrieved from mouse cDNA. GFP-Syt1, GFP-Syt1C2A (1-263aa), and GFP-Syt1C2B (264–422 aa) were constructed by subcloning the correspondent coding region into pEGFP-C1 vector, respectively. GST-tagged Syt1 or its truncated mutant plasmids were generated by subcloning the correspondent coding region into pGEX- 4T-1 vector, respectively. His-Rab26 and DsRed-preproinsulin was contructed by similar method into pet-28a and pDSred-N1 plasmid. GFP-SNAP25 and GFP-Syntaxin1A were from Wanjin Hong’s Laboratory (Institute of Molecular and Cell Biology, Singapore).

Adenovirus was produced as described [51]. Briefly, Rab26WT, Rab26Q123L, and Rab26T77N coding regions were cloned into pAdTrack-CMV vector. The plasmids were then linearized and transformed into the competent AdEasier *E*. *coli* cells to generate recombinant adenovirus plasmid. The recombinant adenovirus plasmids were transfected into 293A cells to produce recombinant adenovirus (referred as Ad-Rab26, etc.).

For lentivirus-mediated protein expression, GFP-Rab26, GFP-Rab26 was cut out from EGFP-C1 vector with NheI/BamH1 and subcloned in the same enzyme sites of pCDH-CMV-MCS-EF1-Puro vector; Syt-1 was subcloned into pCDH-CMV-MCS-EF1-Puro vector using the same approach. For virus preparation, 293T cells were transfected with lentiviral skeleton and helper plasmids (pMD2.G, psPAX2) for 48 h, and the culture medium was collected. In case of virus infection, the cells were inoculated on a 6-well plate, 1 ml of lentivirus stock solution was added after 24 h, and fresh culture medium was changed for 12 h. The expression level of the target protein was detected by western blot.

### CRISPR/Cas9-mediated gene knockout

Rab26-deficient INS-1 cells were generated using CRISPR/Cas9 system as described [18]. sgRNA-1 sequence (5-GTCCTGGGATGTGCCAGACG-3) and sgRNA-2 sequence (5- GAGGGCCGGCCGGACTGCGG -3) were used to disrupt the expression of Rab26 in INS-1 cells. The disruption of Rab26 gene was verified by genomic DNA sequencing and western blot.

### Detection of insulin secretion

MIN6 and INS-1 cells were infected with adenovirus. Cells were preincubated with Krebs–Ringer bicarbonate buffer (KRBH containing 114 mM NaCl, 4.7 mM KCl, 1.2 mM KH_2_PO_4_, 1.16 mM MgSO_4_, 0.5 mM MgCl_2_, 2.5 mM CaCl_2_, 0.25% BSA, and 20 mM HEPES (pH 7.4)) containing 2.8 mM glucose for 60 min, followed by incubation in 2.0 ml of stimulation medium (KRBH containing 16.7 mM glucose). Insulin secretion were detected by ELISA kits (ImmunoDiagnostics Limited, China) at different time points as described [52].

For detection of insulin secretion from islets, islets were isolated from mouse pancreas as described [53], 100 freshly isolated islets or adenovirus-infected islets were applied for perifusion culture under glucose stimulation, the media was collected for insulin measurement by ELISA kits (ImmunoDiagnostics Limited, China) as described above.

### Animal experiments

The body weight of mice was monitored every week, and the blood glucose level was determined by an Accu-Chek glucose meter (Roche Diagnostics). For insulin tolerance tests (ITTs), fasting mice were intraperitoneally injected with 0.75 units of insulin (Gansulin R) per kg body weight, and blood glucose was measured at the designated time points. IPGTT were performed via intraperitoneal injection of 2.0 g/kg glucose into mice after fasting for 16 h as described [54]. Blood samples were taken from the tail vein at the indicated time points.

Type 1 diabetic mice were generated as described [55]. Briefly, mice were intraperitoneally injected with 70 mg/kg streptozotocin (STZ) for 5 days. After a week, the blood glucose level of mice was randomly monitored, and mice with a blood glucose that levels up to 16.7 mM showed that the model of type 1 diabetes (T1DM) was successfully constructed as described [55]. The diabetic mice were transplanted with the freshly isolated islets. About 300 islets infected by Ad-Rab26 or Ad-vector were suspended in 20 μl RPMI medium containing 10% FBS. Then, the virus-infected islets were transplanted into the renal capsule of mice as described [56]. IPGTT was generated as described above.

Male mice at 6 to 8 wk old were used for the investigation. Mice were exposed to light/dark cycle for 12 h at room temperature with sufficient food and water. Rab26^-/-^ mice were bred by heterozygosity and maintained in the Xiamen University Laboratory Animal Center. BKS db/db diabetic mice were obtained from Model Animals Research Center of Nanjing University (#T002407, BKS-lepr^em2Cd479^/Nju), which is derived from BSK gene background with nutation of leptin receptor. All animal experiments were conducted in strict accordance with the guiding principles of experimental animals in the regulations of Institutional Animal Ethics Committee of Xiamen University with experimental animal ethics approval No.XMULAC20180125.

### Immunofluorescence microscopy

Immunostaining was performed as previously described [57]. Briefly, cells grown on coverslips were washed with PBSCM (PBS containing 1.0 mM CaCl2 and 1.0 mM MgCl2) for 10 min 3 times and then fixed with 4% paraformaldehyde at room temperature for 20 min. After 3 times of PBSCM washing, cells were permeabilized with 0.1% Triton-100 (Sigma) in PBSCM for 10 min at room temperature.

For tissue staining, the islets were cryosectioned, and the cryosections were permeabilized with 0.2% Triton X-100 and then blocked with 0.2% BSA and subjected for immunostaining using the primary antibodies, followed by fluorophone-conjugated secondary antibodies. The immunolabeled cells or tissues were analyzed with Carl Zeiss LSM7 EXITER or Leica TCS SP8 STED laser scanning confocal microscope.

### Immunohistological chemistry

Pancreatic tissue was immunohistochemical stained to detect the expression profile of Rab26 according the manufacturer’s protocol. Briefly, the paraffin-embedded pancreatic tissues were sectioned to a thickness of 4-μm deparaffinized and rehydrated, then processed for antigen recovery, after blocking with 3% BSA for 30 min at room temperature, and then incubated with Rab26 antibody and treated with HRP-conjugated secondary antibody (ZSGB-BIO, cat.No.PV-9001), then incubated with DAB for color development. The tissue was observed under microscope (Olympus BX53).

### Co-immunoprecipitation, GST-pulldown assay, and western blot

Co-immunoprecipitation assays and GST-pulldown assay were applied for detection of protein–protein interaction. Cells were lysed with 1 ml lysis buffer (containing 20 mM HEPES (pH 7.4), 1% Triton-100, 100 mM NaCl, 5 mM MgCl2, and EDTA-free proteinase inhibitor cocktail from Roche) for 1 h on ice. The cell lysates were clarified by spinning at 14,000 rpm for 30 min.

For co-immunoprecipitation assay, INS-1 cells lysates were immunoprecipitated with anti-Syt1 antibody. For GST-pulldown assay, 293T cells transfected with the indicated plasmids. The supernatants were incubated with GST-fusion protein coupled to GST-Sepharose 4B resin (GE Healthcare, cat. No. 45-000-139) at 4°C for 4 h. The proteins bound to GST sepharose beads were detected through western blot assay.

Western blot was performed as described [58]. Briefly, cells were lysed in RIPA buffer containing proteinase inhibitor cocktail (Roche, cat. no. 04693132001). The resulted cell lysates were separated by SDS-PAGE, the resolved proteins were transferred to PVDF membrane, and then the membrane was blocked with 5% milk in TBST, followed by incubation with primary antibodies and HRP-conjugated secondary antibody. The blots were detected using ECL system (Pierce, Rockford, IL, USA).

### TIRF microscopy and data analysis

MIN6 cells were cotransfected with DsRed-preproinsulin and Vector or GFP-Rab26 for 24 h, respectively. Before image acquisition, the cells were preincubated for 30 min in KRB buffer containing 2.8 mM glucose. The microscope is equipped with a temperature control device and a carbon dioxide device to keep the experiment at 37°C with 5% carbon dioxide. The cells were incubated with 2.8 mM glucose for 2 min, then 2.8 mM glucose containing 10 nM GLP-1 and 150 μM IBMX (Isobutyl-1-methylxanthine) for 3 min, and finally stimulated with 16.7 mM glucose containing 10 nM GLP-1 and 150 μM IBMX for 18 min.

Time-lapse TIRF microscopy was performed on a Nikon Ti-E inverted microscope using a 100× oil immersion TIRF objective (NA 1.49). We used an Agilent MLC-400B laser launch with 488 nm and 568 nm solid-state lasers to excite EGFP and DsRed, respectively. The fluorescence signals were detected by an electron-multiplying charge-coupled device camera (EMCCD) under the control of NIS-Elements AR software. TIRF images were acquired at 5 Hz with an exposure time of 100 ms. SGs were localized, counted, and analyzed using ImageJ and Imaris.

Fusion events, observed as flashes of fluorescence indicating emptying of DsRed-preproinsulin cargo, were manually selected, as recently reported in detail [59]. An increase of DsRed fluorescence exceeding 5 times over the standard deviation of the fluorescence fluctuation was considered as fusion events. We used a concentric circle (approximately 7 pixels with a pixel size of 160 nm, corresponding to approximately 1.12 μm diameter) to center on the selected SGs to characterize the evolution of fluorescence over time of single SGs on background-subtracted images. Fusion events were also indicated by abrupt brightening of fluorescence and were manually selected for analyses of SGs exocytosis.

### Transmission electronic microscopy

MIN6 cells were infected with Ad-Vector or Ad-Rab26. Cells were processed for transmission electronic microcopy analysis as described [18]. The ultrasectioned samples were analyzed using transmission electron microscope (HT7800 RuliTEM). Morphologically docked granules were defined using the criteria previously described [60]. All granules in β cells were examined, and cytoplasm area, granule number, and distance from plasma membrane were measured using ImageJ software.

### Statistical analysis

The data were presented as mean ± standard deviation (SD) with GraphPad Prism 7.0. Statistical significance between 2 groups was assessed using Student *t* tests or among multiple groups using two-way ANOVA. A value of *P* < 0.05 was indicated as statistical significance.

## Supporting information

S1 Fig (A) mRNA level of Rab26 in different tissues of WT mice with RT-PCR. (B) Rab26 protein expression in β cells and PC12 cells at the protein level. Western blot was used to detect Rab26 and α-tubulin. (C) Frozen sections of pancreas from WT and Rab26^-/-^ mice, insulin antibody, and DAPI immunofluorescence staining showed no obvious morphological changes in the islets. (D) Islets were incubated for 10 min with 2.8 mM glucose; the secreted insulin was detected by ELISA. (E) and (F) mRNA levels in INS-1 cells were detected by agarose electrophoresis and qPCR, indicating that Rab26 KO did not affect the transcription of insulin1 (ins1), insulin2 (ins2), and preproinsulin. Primers were listed in S1 Table. (G) Body weight of WT or KO mice were measured in the morning. The numerical values that were used to generate graphs and histograms can be found in S1 Data. KO, knockout; RT-PCR, reverse transcription PCR; WT, wild-type.
(TIF)

S2 Fig (A) MIN6 cells were effectively infected by recombinant adenovirus expressing Rab26 (Ad-Rab26). (B) Adenovirus mediated overexpression of Rab26 detected by western blot in MIN6 cells. (C) Cells were incubated for 10 min with 2.8 mM glucose; the secreted insulin was detected by ELISA. (D, E) Overexpression of Rab26 in INS-1 cells and detection of mRNA levels of ins1, ins2, and preproinsulin by agarose electrophoresis and qPCR. Primers were mentioned in S1 Fig. (F) Adenovirus mediated overexpression of Rab26, Rab26T77N, or Rab26Q123L were detected by western blot in INS-1 cells. (G, H) Freshly isolated islets from WT mouse or Rab26^-/-^ mice were infected with Ad-Rab26 or Ad-vector and assessed by fluorescence microscopy and western blot. The numerical values that were used to generate graphs and histograms can be found in S1 Data.
(TIF)

S3 Fig (A) Mouse liver lysate was subjected for large-scale pulldown experiments using GST- Rab26QL, Rab26TN, and Rab26WT. Silver staining is used to identify the interaction proteins after SDS-PAGE. Multiple interactive proteins were detected by liquid chromatography-mass spectrometry (LC-MS). (B) 293t cells were transfected with GFP-Syt1, Syt4, and Syt7 respectively; cell lysates were processed for GST-pulldown with GST-Rab26, and the results demonstrated that Rab26 does not interact with either Syt4 or Syt7. (C) Cell lysates containing GFP-Syt1 or GFP-Syt2 were subjected for western blot; GFP antibody can recognize both proteins, but Syt1 antibody only recognize GFP-Syt1, not GFP-Syt2. (D) 293t cells were transfected with GFP-Syt1; cell lysates were processed for GST-pulldown experiment with GST-Rab26 or GST-Rab26b and analyzed by western blot with GFP antibody. (E) 293t cells were transfected with GFP-Vector, GFP-Rab26, Rab3, and Rab27 respectively; cell lysates were processed for GST-pulldown with GST-Syt1 and analyzed by western blotting with GFP antibody.
(TIF)

S4 Fig (A) INS-1 cells were infected with Ad-vector or Ad-Rab26, after 48 h, and INS-1 cells were grown in complete normal medium (Medium), containing 16.7 mM glucose medium (16.7 mM) or 2 mM Ca^2+^ medium (2 mM Ca^2+^) for 24 h. Western blot was used to detect Syt1, SNAP25, Syntaxin-1, Rab26, and α-tubulin. (B) Lipid-protein overlay assay was performed by using PIP Strips membranes (P23751, Invitrogen) according to the manufacturer’s instructions, showing Rab26 not influencing Syt1 binding to Ptdlns(4,5)P_2_ and PS.
(TIF)

S5 Fig (A)Western blot for LC3 from INS-1 cells were infected with Ad-vector or Ad-Rab26, after 48 h, and INS-1 cells in nutrient starvation medium for 0 h, 2 h, 4 h, or 8 h. Then, western blot was used to detect LC3, Rab26, and α-tubulin. (B) Quantitative analysis of the results of A from 3 independent experiments. (C) INS-1 cells were infected with Ad-vector, Ad-Rab26WT, Ad-Rab26TN, or Ad-Rab26QL, after 48 h, and INS-1 cells in nutrient starvation medium for 0 h or 8 h. Western blot was used to detect LC3, Rab26, and α-tubulin. (D) Quantitative analysis of the results of C from 3 independent experiments. (E) INS-1 cells were infected with Ad-vector or Ad-Rab26, after 48 h, and INS-1 cells in normal complete medium (Medium), nutrient starvation medium (No AA), or glucose-free medium (No Glc) for 8 h. Western blot was used to detect LC3, Rab26, and α-tubulin. (F) Quantitative analysis of the results of E from 3 independent experiments. The numerical values that were used to generate graphs and histograms can be found in S1 Data.
(TIF)

S6 Fig qPCR approach was used to detect the mRNA levels of Rab3a, Rab27a, Munc18a, b, Vamp2, Syntaxin 1A, Syntaxin 3, Syt1, or SNAP25. The primers were listed in S1 Table. The numerical values that were used to generate graphs and histograms can be found in S1 Data.
(TIF)

S1 Table Primer sequence information used in this study. (XLSX)

S1 Raw Images Uncropped western blots, gel blots for Figs 1B, 1C, 1F, 3L, 3N, 5A, 5B, 5C, 5D, 6A, 6C, 6E, 6H, 7A, 7C, 7E, S1A, S1B, S1E, S2B, S2D, S2F, S2H, S3A, S3B, S3C, S3D, S3E, S4A, S5A, S5C and S5E. (PDF)

S1 Data Numerical data for Figs 1D, 1E, 1G, 1H, 1I, 1J, 1K, 1L, 1M, 1N, 2A, 2B, 2C, 2D, 2E, 2F, 2G, 2H, 3B, 3C, 3D, 3G, 3H, 3I, 3J, 3M, 3O, 6B, 6D, 6F, 6G, 6I, 7B, 7D, 7F, 8A, 8B, 8C, 8D, 8F, S1D, S1F, S1G, S2C, S2E, S5B, S5D, S5F and S6. (XLSX)

## Abbreviations

GOMED: Golgi membrane-associated degradation
GSIS: glucose-stimulated insulin secretion
IPGTT: intraperitoneal glucose tolerance test
IPITT: intraperitoneal insulin tolerance test
ISG: insulin secretory granule
ITT: insulin tolerance test
KO: knockout
mAb: monoclonal antibody
RILP: Rab7-interacting lysosomal protein
SINGD: starvation-induced nascent granule degradation
Syt1: Synaptotagmin-1
TIRFM: total internal reflection fluorescence microscopy
WT: wild-type
