# Supplementary figures and images for: Rab26 restricts insulin secretion via sequestering Synaptotagmin-1
Source: PLoS Biol. 2023 Jun 8;21(6):e3002142. doi: 10.1371/journal.pbio.3002142 (PMC10284394; doi:10.1371/journal.pbio.3002142)

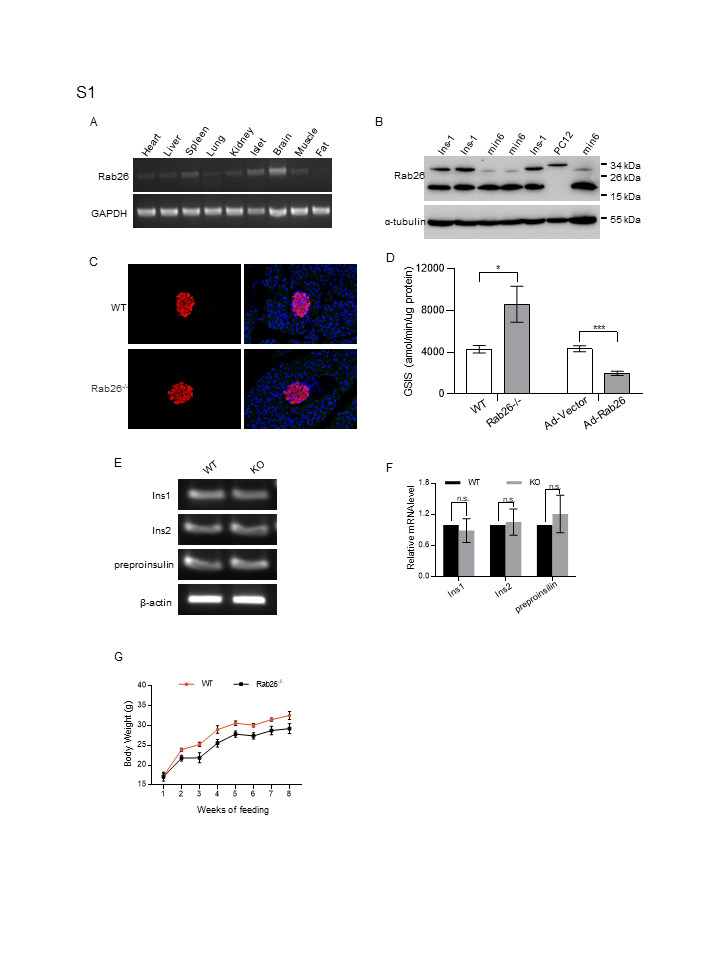

Supplement: S1 Fig — (A) mRNA level of Rab26 in different tissues of WT mice with RT-PCR. (B) Rab26 protein expression in β cells and PC12 cells at the protein level. Western blot was used to detect Rab26 and α-tubulin. (C) Frozen sections of pancreas from WT and Rab26-/- mice, insulin antibody, and DAPI immunofluorescence staining showed no obvious morphological changes in the islets. (D) Islets were incubated for 10 min with 2.8 mM glucose; the secreted insulin was detected by ELISA. (E) and (F) mRNA levels in INS-1 cells were detected by agarose electrophoresis and qPCR, indicating that Rab26 KO did not affect the transcription of insulin1 (ins1), insulin2 (ins2), and preproinsulin. Primers were listed in S1 Table. (G) Body weight of WT or KO mice were measured in the morning. The numerical values that were used to generate graphs and histograms can be found in S1 Data. KO, knockout; RT-PCR, reverse transcription PCR; WT, wild-type. (TIF) [file pbio.3002142.s001.TIF]

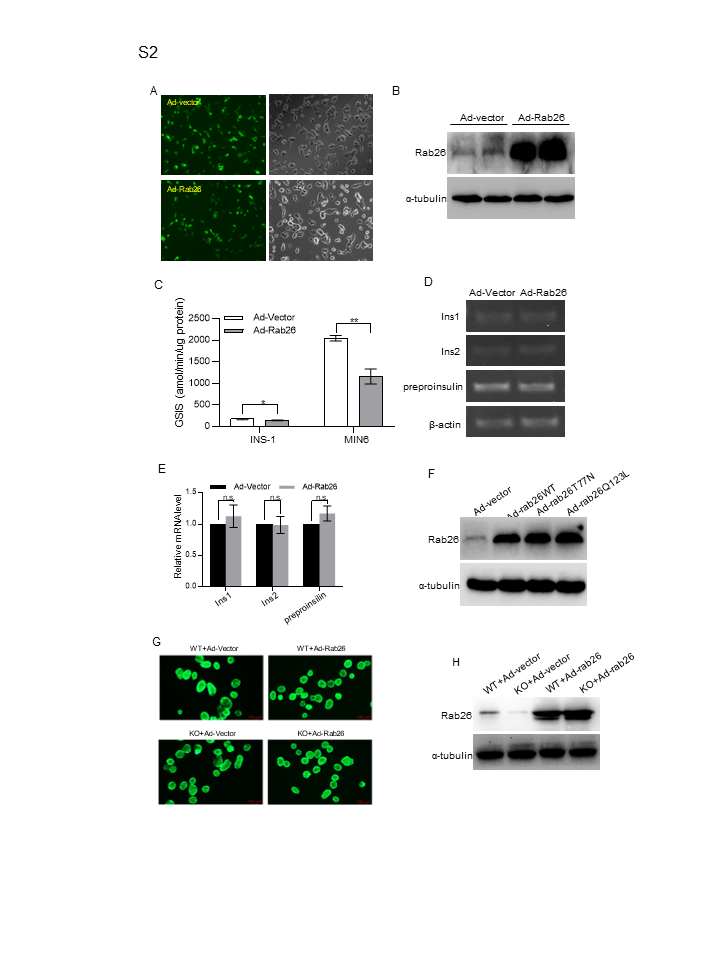

Supplement: S2 Fig — (A) MIN6 cells were effectively infected by recombinant adenovirus expressing Rab26 (Ad-Rab26). (B) Adenovirus mediated overexpression of Rab26 detected by western blot in MIN6 cells. (C) Cells were incubated for 10 min with 2.8 mM glucose; the secreted insulin was detected by ELISA. (D, E) Overexpression of Rab26 in INS-1 cells and detection of mRNA levels of ins1, ins2, and preproinsulin by agarose electrophoresis and qPCR. Primers were mentioned in S1 Fig. (F) Adenovirus mediated overexpression of Rab26, Rab26T77N, or Rab26Q123L were detected by western blot in INS-1 cells. (G, H) Freshly isolated islets from WT mouse or Rab26-/- mice were infected with Ad-Rab26 or Ad-vector and assessed by fluorescence microscopy and western blot. The numerical values that were used to generate graphs and histograms can be found in S1 Data. (TIF) [file pbio.3002142.s002.TIF]

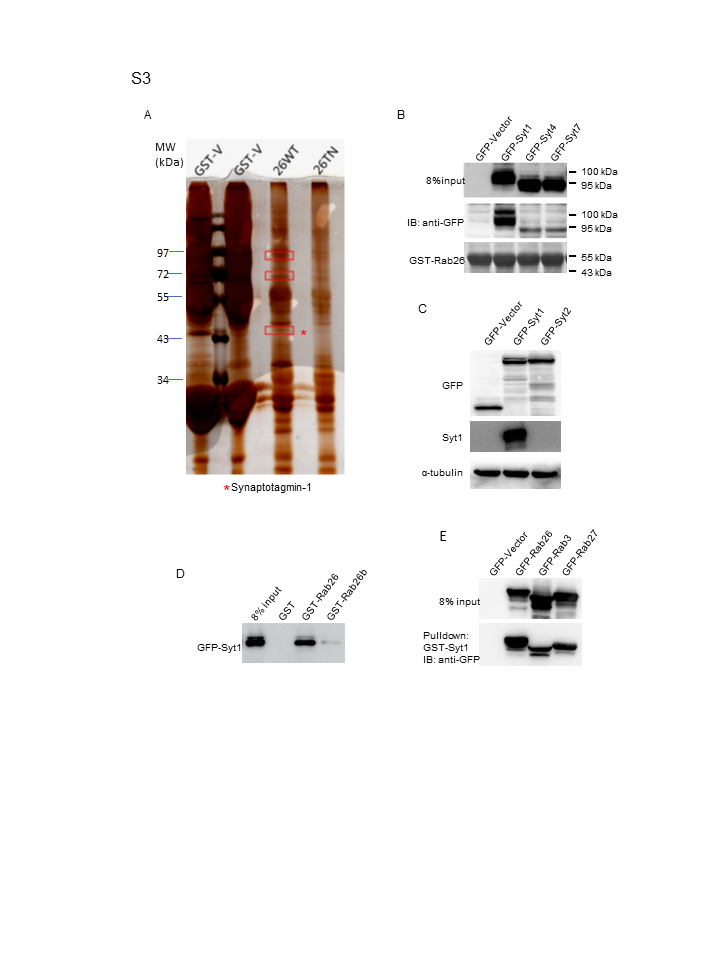

Supplement: S3 Fig — (A) Mouse liver lysate was subjected for large-scale pulldown experiments using GST- Rab26QL, Rab26TN, and Rab26WT. Silver staining is used to identify the interaction proteins after SDS-PAGE. Multiple interactive proteins were detected by liquid chromatography-mass spectrometry (LC-MS). (B) 293t cells were transfected with GFP-Syt1, Syt4, and Syt7 respectively; cell lysates were processed for GST-pulldown with GST-Rab26, and the results demonstrated that Rab26 does not interact with either Syt4 or Syt7. (C) Cell lysates containing GFP-Syt1 or GFP-Syt2 were subjected for western blot; GFP antibody can recognize both proteins, but Syt1 antibody only recognize GFP-Syt1, not GFP-Syt2. (D) 293t cells were transfected with GFP-Syt1; cell lysates were processed for GST-pulldown experiment with GST-Rab26 or GST-Rab26b and analyzed by western blot with GFP antibody. (E) 293t cells were transfected with GFP-Vector, GFP-Rab26, Rab3, and Rab27 respectively; cell lysates were processed for GST-pulldown with GST-Syt1 and analyzed by western blotting with GFP antibody. (TIF) [file pbio.3002142.s003.TIF]

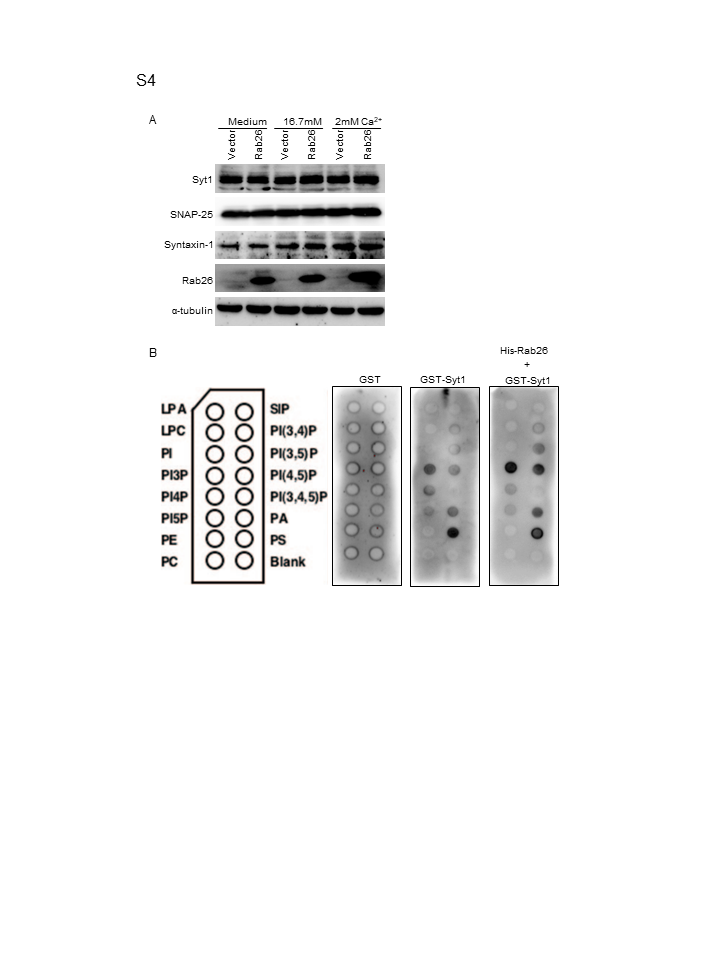

Supplement: S4 Fig — (A) INS-1 cells were infected with Ad-vector or Ad-Rab26, after 48 h, and INS-1 cells were grown in complete normal medium (Medium), containing 16.7 mM glucose medium (16.7 mM) or 2 mM Ca2+ medium (2 mM Ca2+) for 24 h. Western blot was used to detect Syt1, SNAP25, Syntaxin-1, Rab26, and α-tubulin. (B) Lipid-protein overlay assay was performed by using PIP Strips membranes (P23751, Invitrogen) according to the manufacturer’s instructions, showing Rab26 not influencing Syt1 binding to Ptdlns(4,5)P2 and PS. (TIF) [file pbio.3002142.s004.TIF]

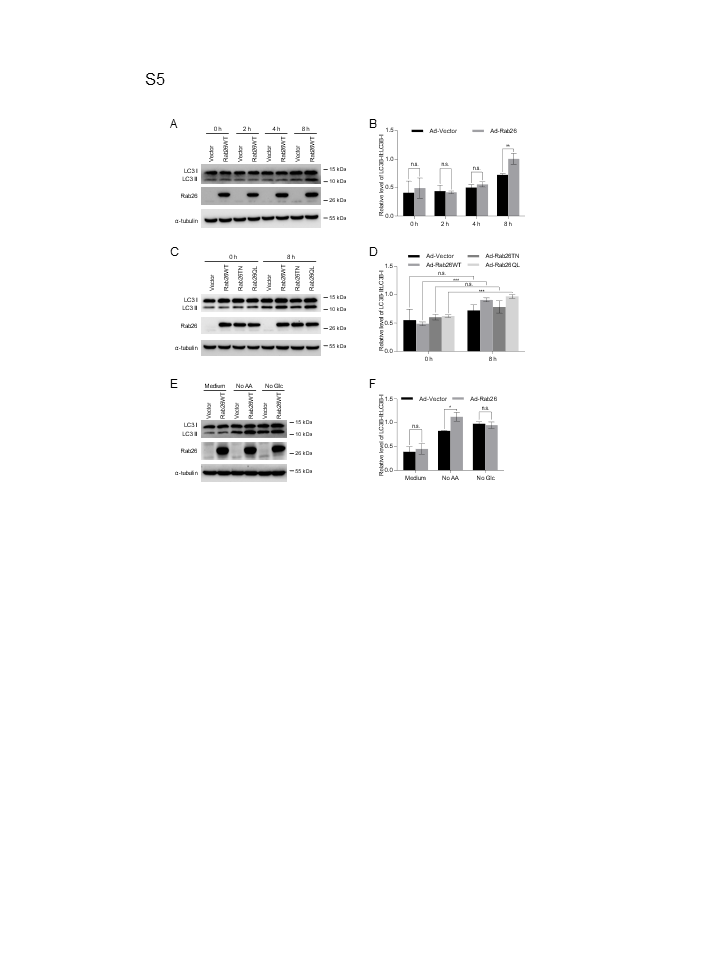

Supplement: S5 Fig — (A)Western blot for LC3 from INS-1 cells were infected with Ad-vector or Ad-Rab26, after 48 h, and INS-1 cells in nutrient starvation medium for 0 h, 2 h, 4 h, or 8 h. Then, western blot was used to detect LC3, Rab26, and α-tubulin. (B) Quantitative analysis of the results of A from 3 independent experiments. (C) INS-1 cells were infected with Ad-vector, Ad-Rab26WT, Ad-Rab26TN, or Ad-Rab26QL, after 48 h, and INS-1 cells in nutrient starvation medium for 0 h or 8 h. Western blot was used to detect LC3, Rab26, and α-tubulin. (D) Quantitative analysis of the results of C from 3 independent experiments. (E) INS-1 cells were infected with Ad-vector or Ad-Rab26, after 48 h, and INS-1 cells in normal complete medium (Medium), nutrient starvation medium (No AA), or glucose-free medium (No Glc) for 8 h. Western blot was used to detect LC3, Rab26, and α-tubulin. (F) Quantitative analysis of the results of E from 3 independent experiments. The numerical values that were used to generate graphs and histograms can be found in S1 Data. (TIF) [file pbio.3002142.s005.TIF]

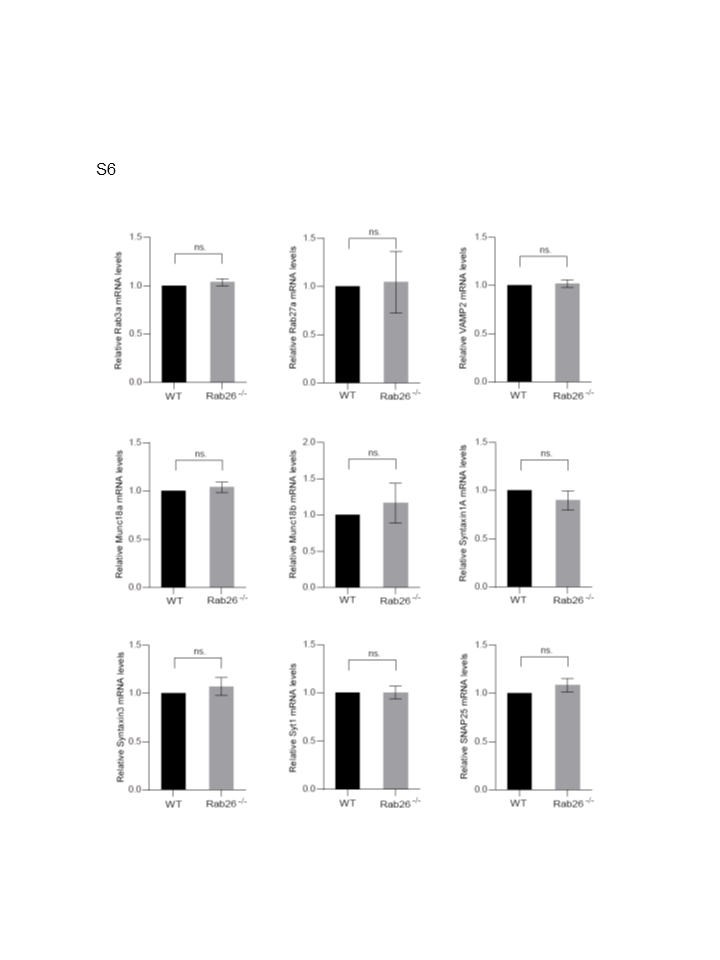

Supplement: S6 Fig — qPCR approach was used to detect the mRNA levels of Rab3a, Rab27a, Munc18a, b, Vamp2, Syntaxin 1A, Syntaxin 3, Syt1, or SNAP25. The primers were listed in S1 Table. The numerical values that were used to generate graphs and histograms can be found in S1 Data. (TIF) [file pbio.3002142.s006.TIF]
