# Supplementary material for: Rab26 restricts insulin secretion via sequestering Synaptotagmin-1
Source: PLoS Biol. 2023 Jun 8;21(6):e3002142. doi: 10.1371/journal.pbio.3002142 (PMC10284394; doi:10.1371/journal.pbio.3002142)

Rab 26 restricts insulin secretion via sequestering Synaptotagmin-1

Ruijuan Zhuang et al.

Uncropped original blots

Fig 1 B

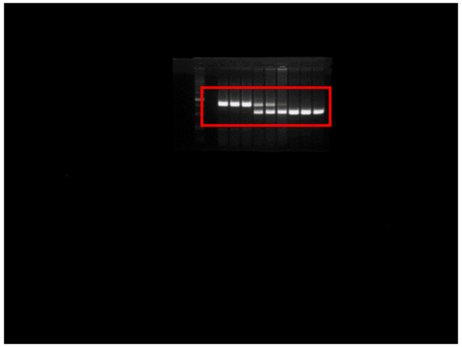

Rab26

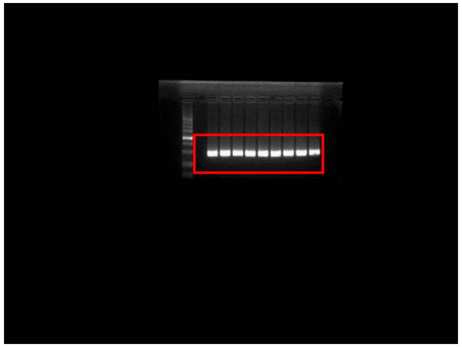

α-tubulin

Fig 1 C

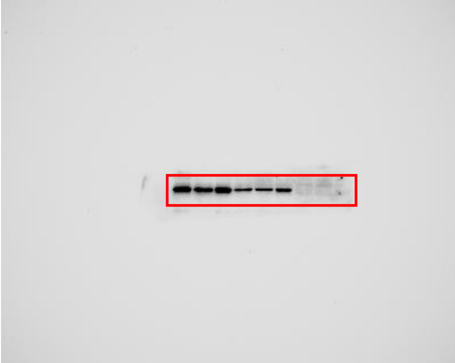

Rab26

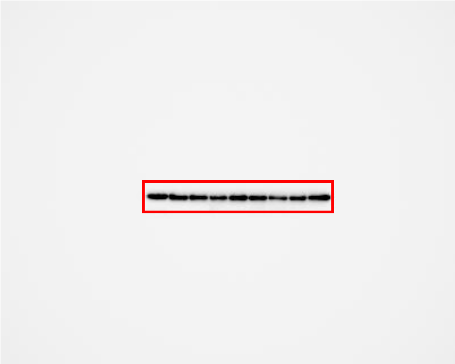

α-tubulin

Fig 1 F

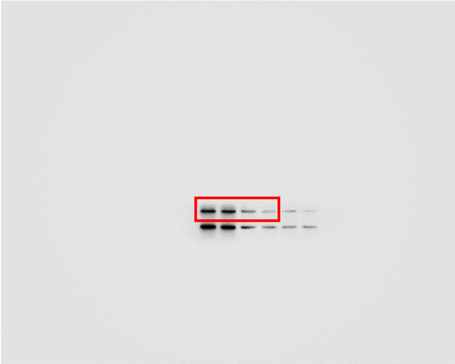

Rab26

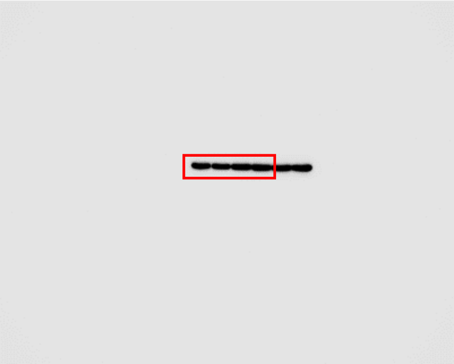

α-tubulin

Fig 3 L

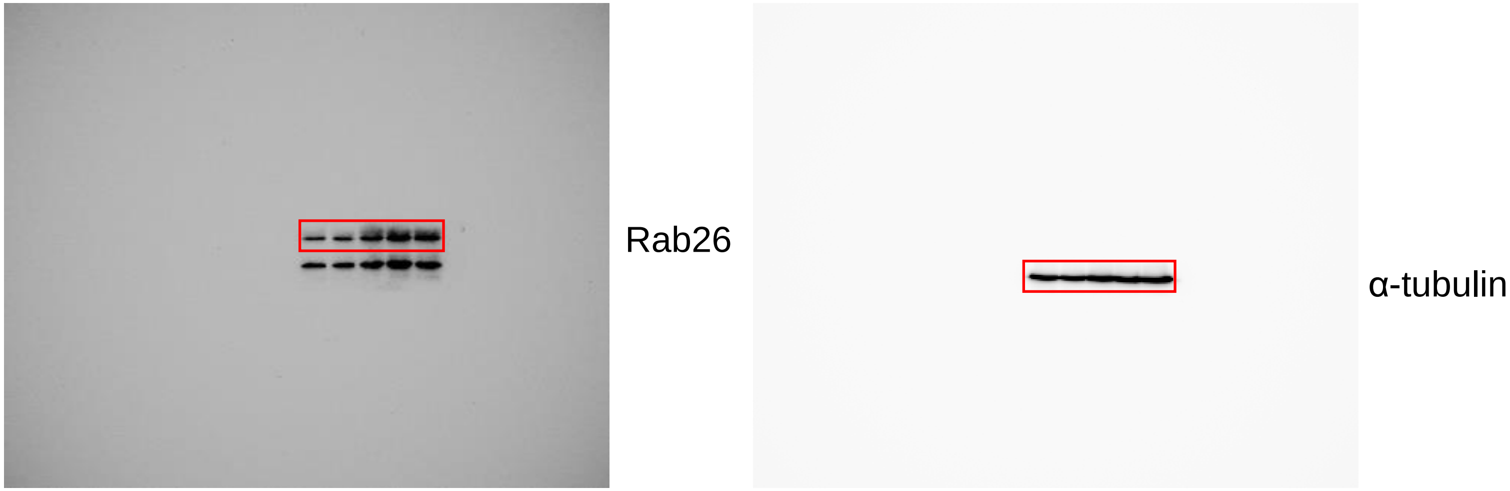

Fig 3 N

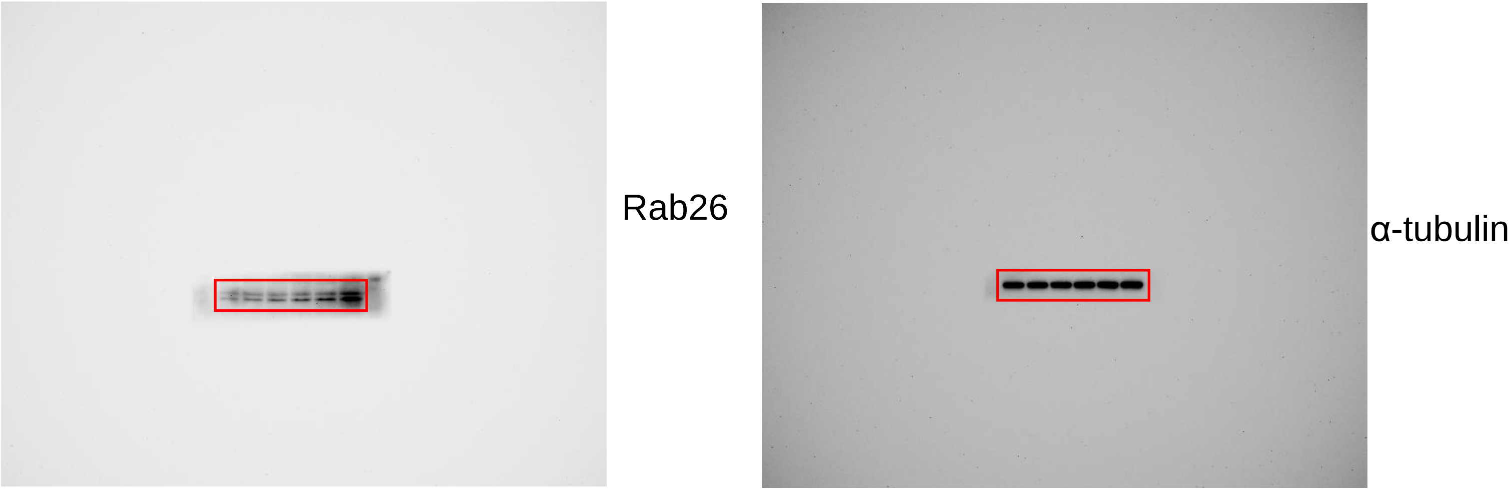

Fig 5 A

Rab26

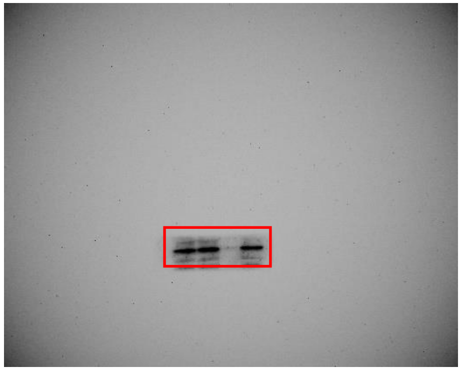

Fig 5 B

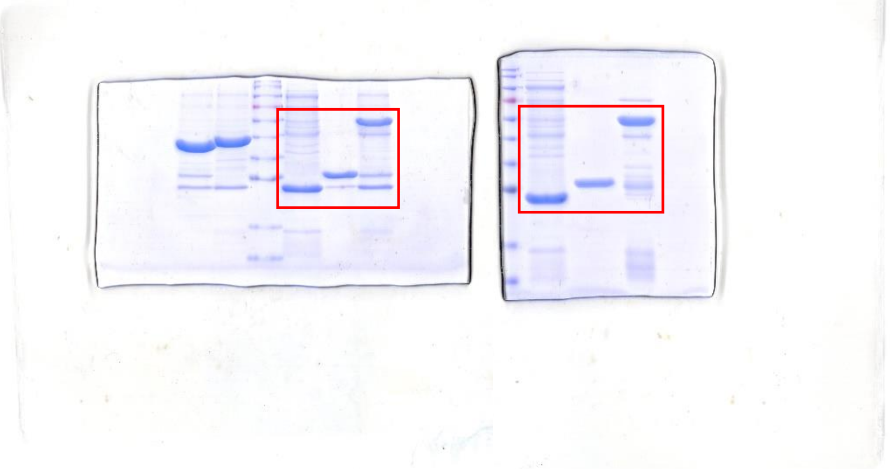

Fig 5 C

GFP-syt1

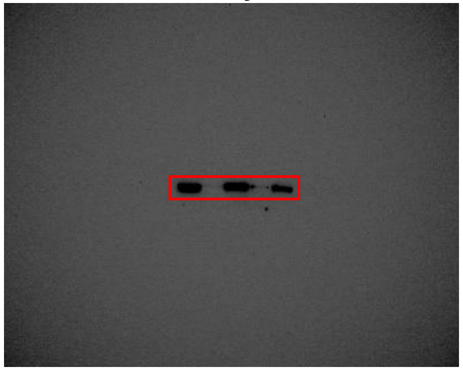

GFP-syt1-C2A

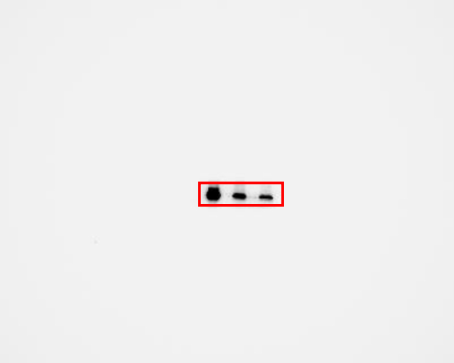

GFP-syt1-C2B

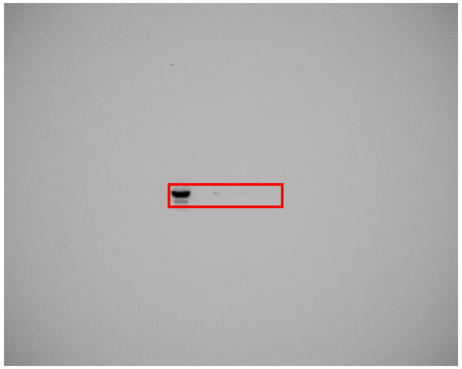

Fig 5 D

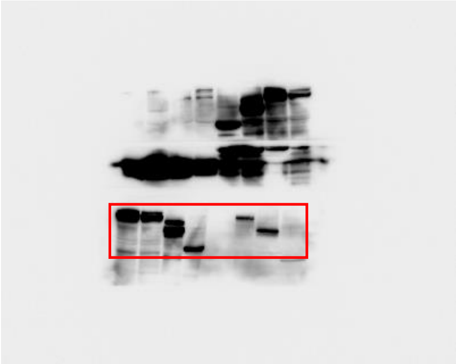

Fig 6A Syt1 Rab26 α-tubulin

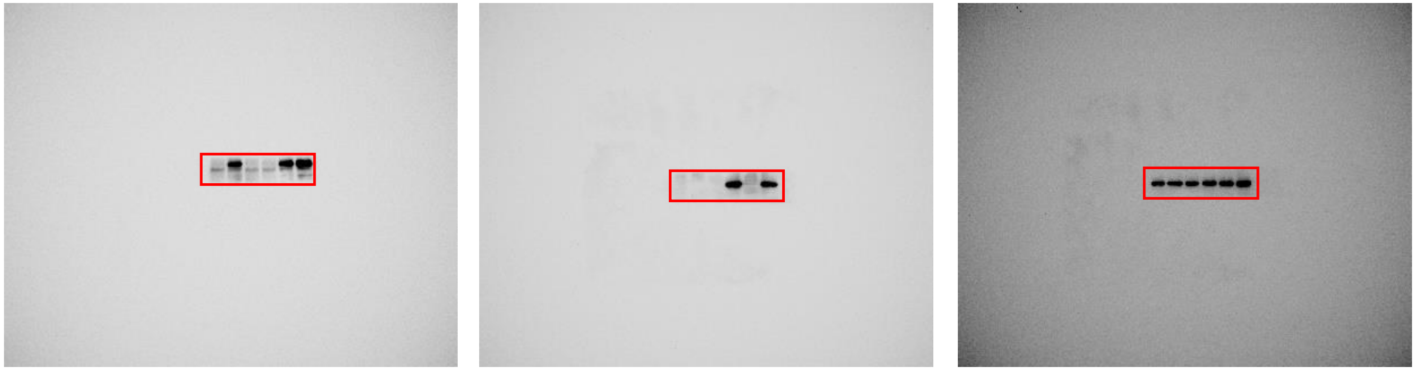

Fig 6C Syt1 Rab26 α-tubulin

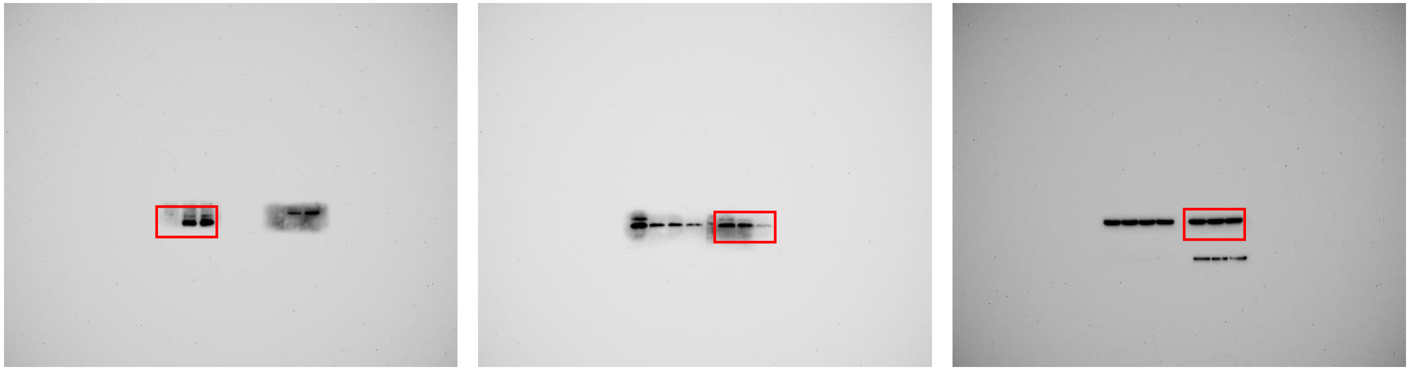

Fig 6E

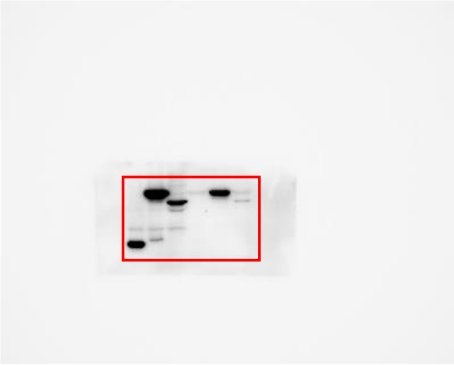

Fig 6H Rab26 Rab26 α-tubulin

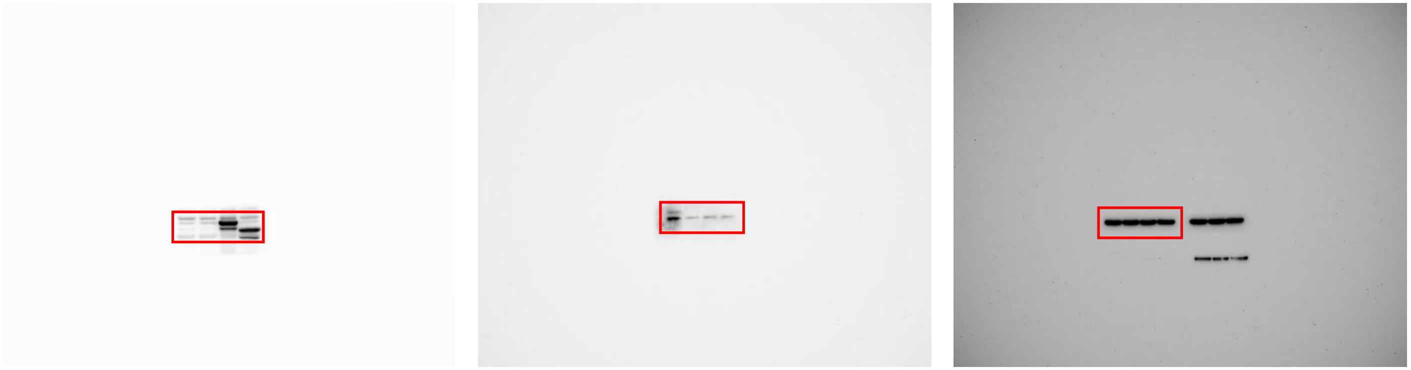

Fig 7A

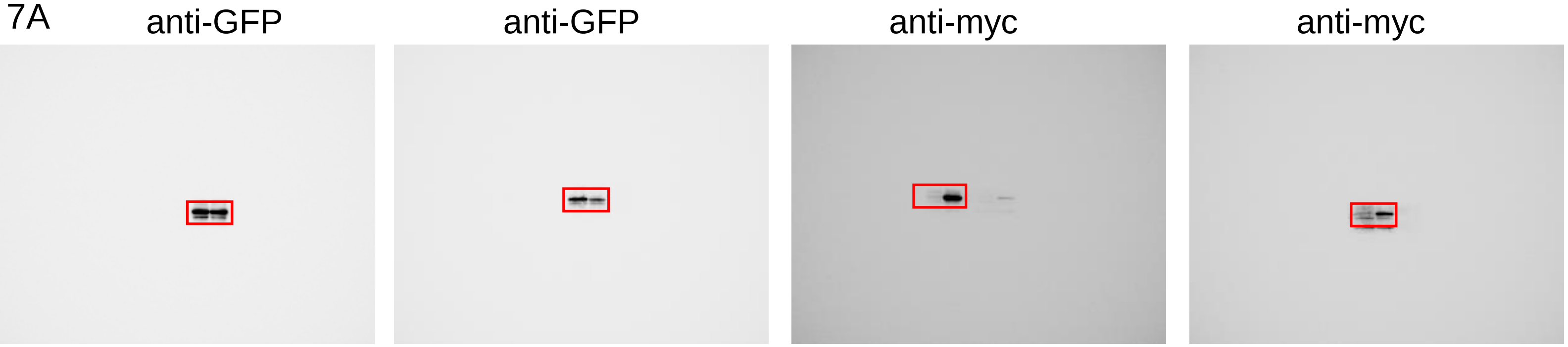

Fig 7C

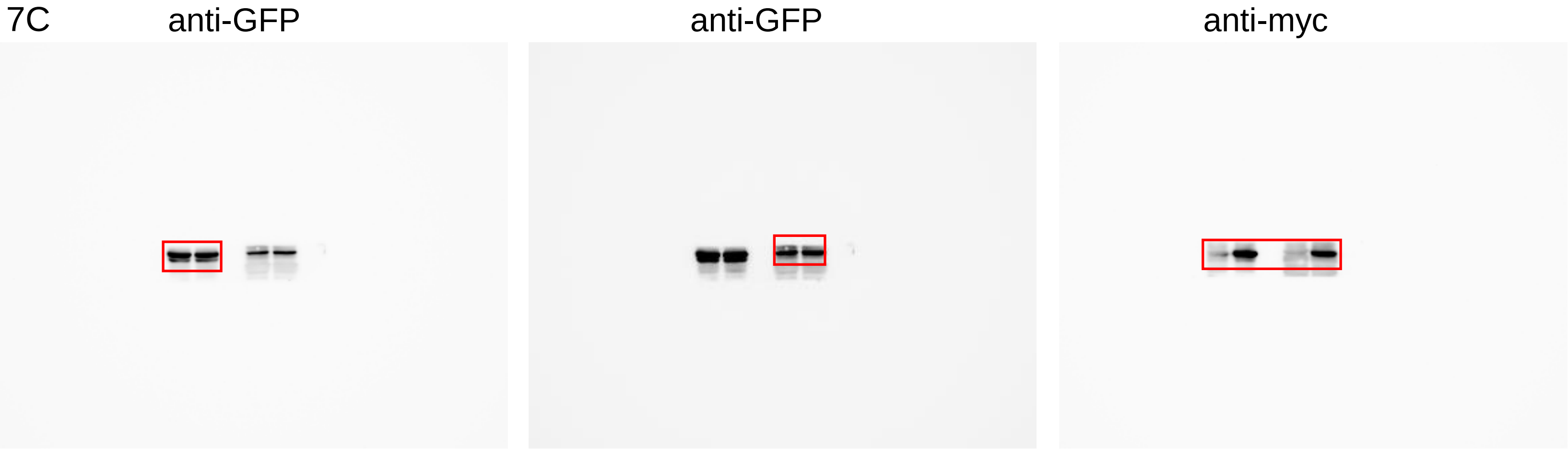

Fig 7E

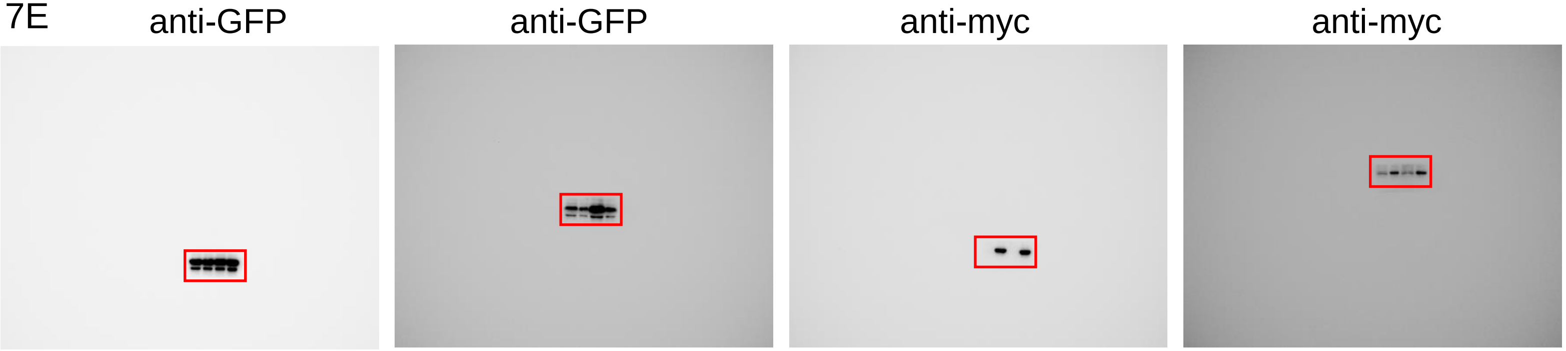

Fig S1A

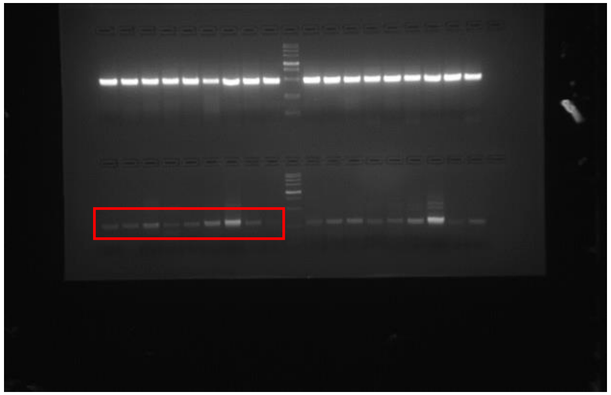

Rab26

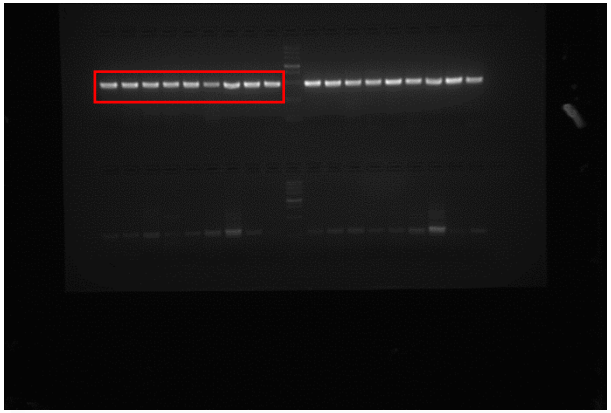

α-tubulin

Fig S1B

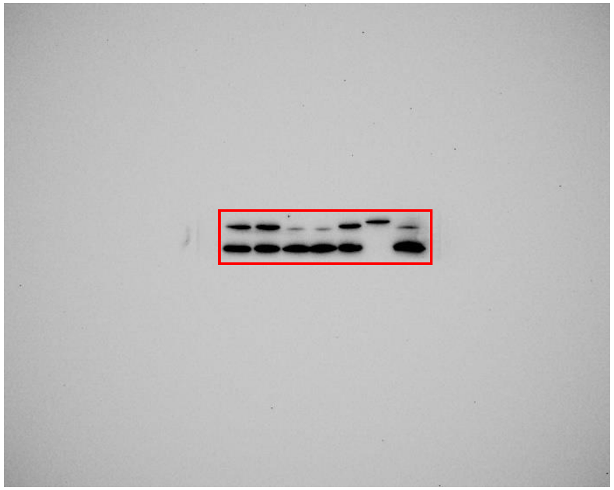

Rab26

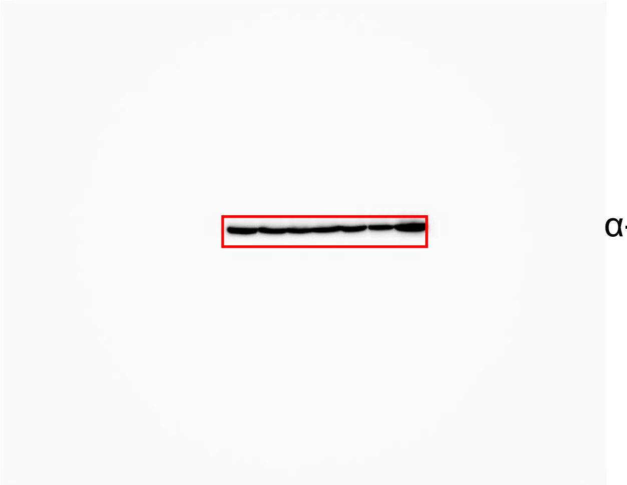

α-tubulin

Fig S1E

Ins1 Ins2 preproinsulin

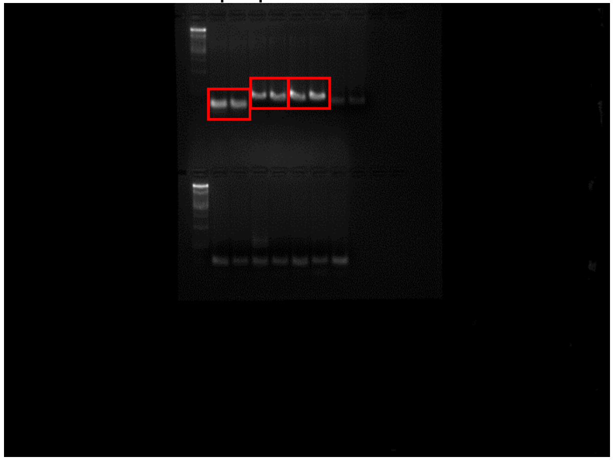

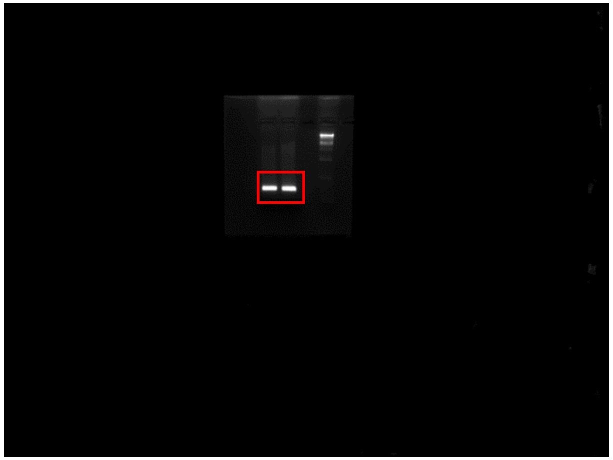

β-actin

Fig S2B

Rab26 α-tubulin

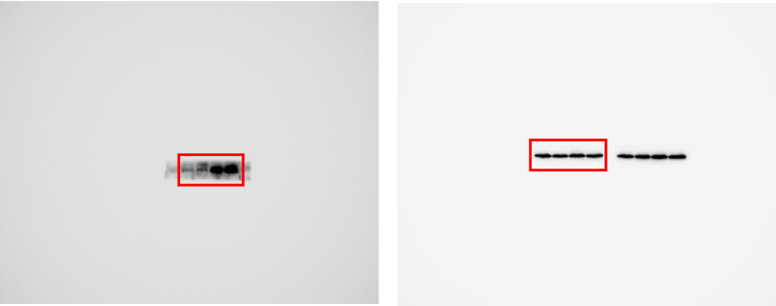

Fig S2D

preproinsulin Ins1 Ins2

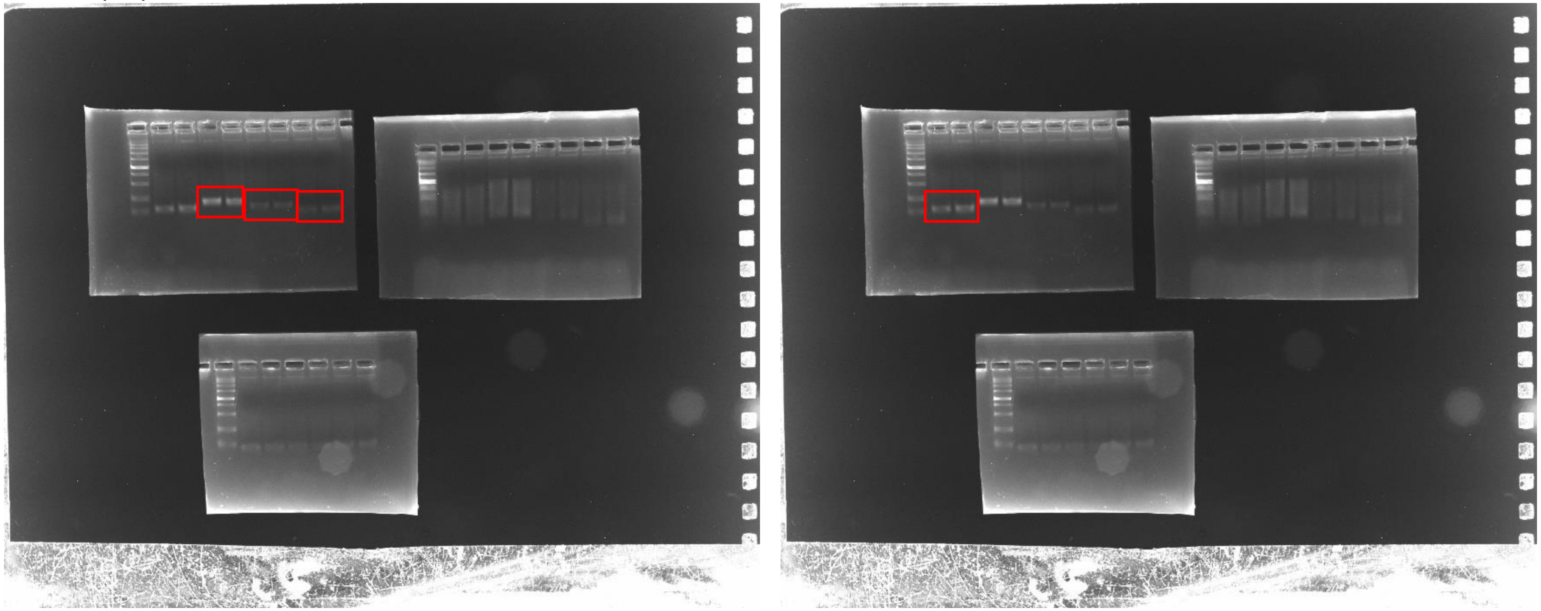

β-actin

Fig S2F

Rab26 α-tubulin

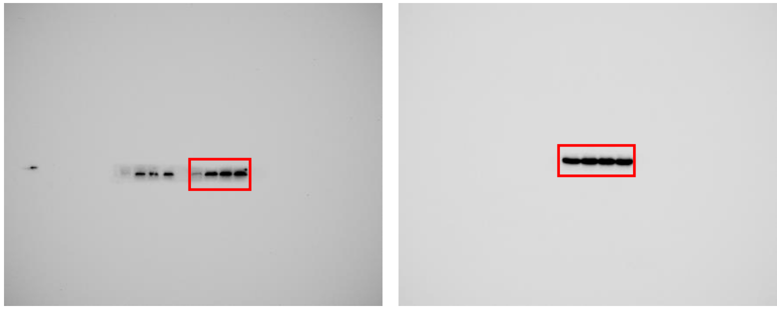

Fig S2H

Rab26 α-tubulin

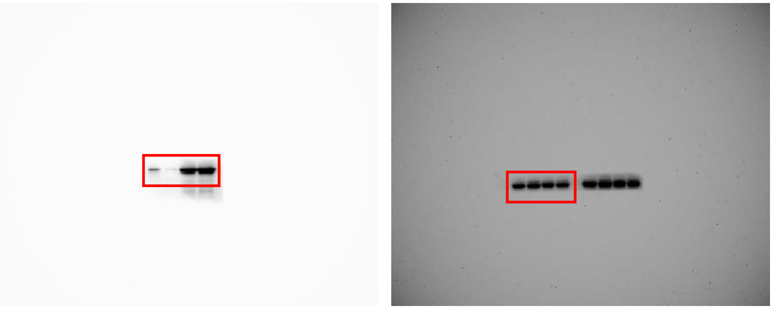

Fig S3A

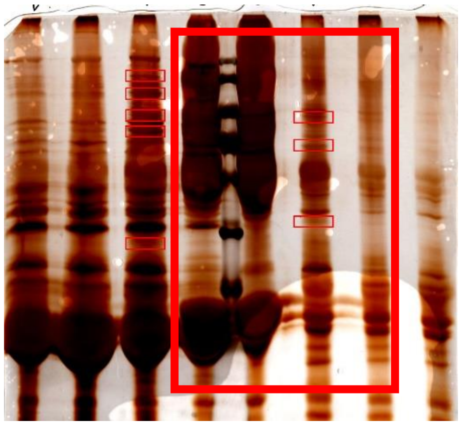

Fig S3B

anti-GFP anti-GFP

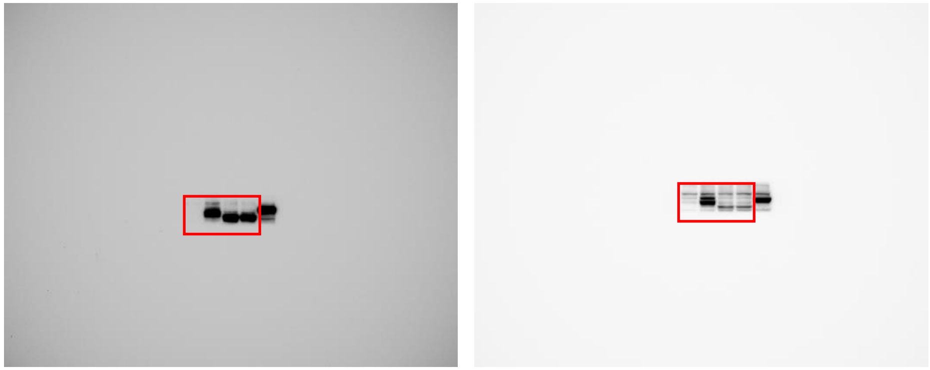

Fig S3C

GFP Syt1 α-tubulin

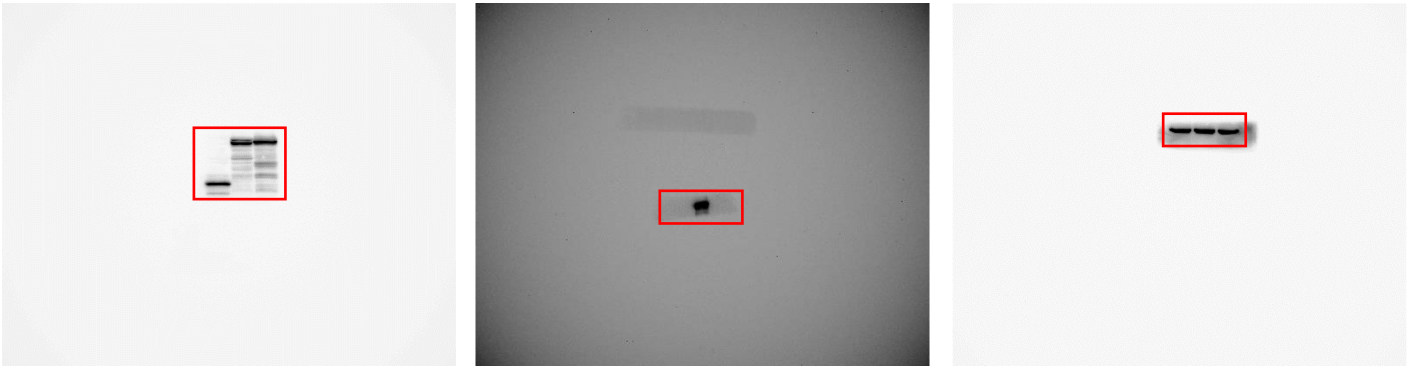

Fig S3D

anti-GFP

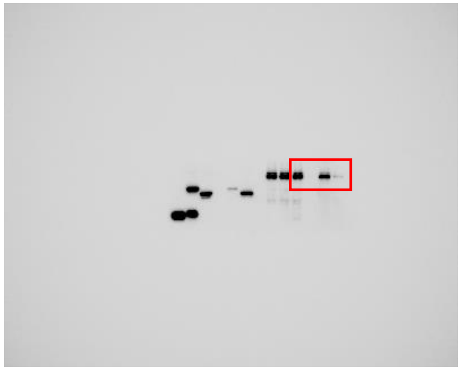

Fig S3E

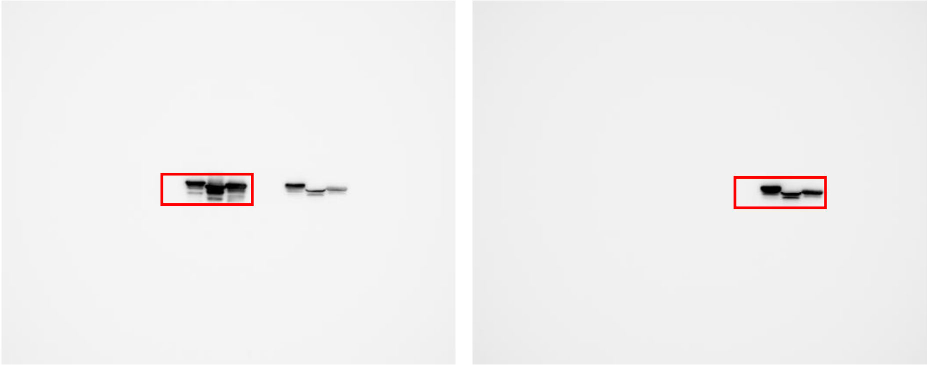

Fig S4A

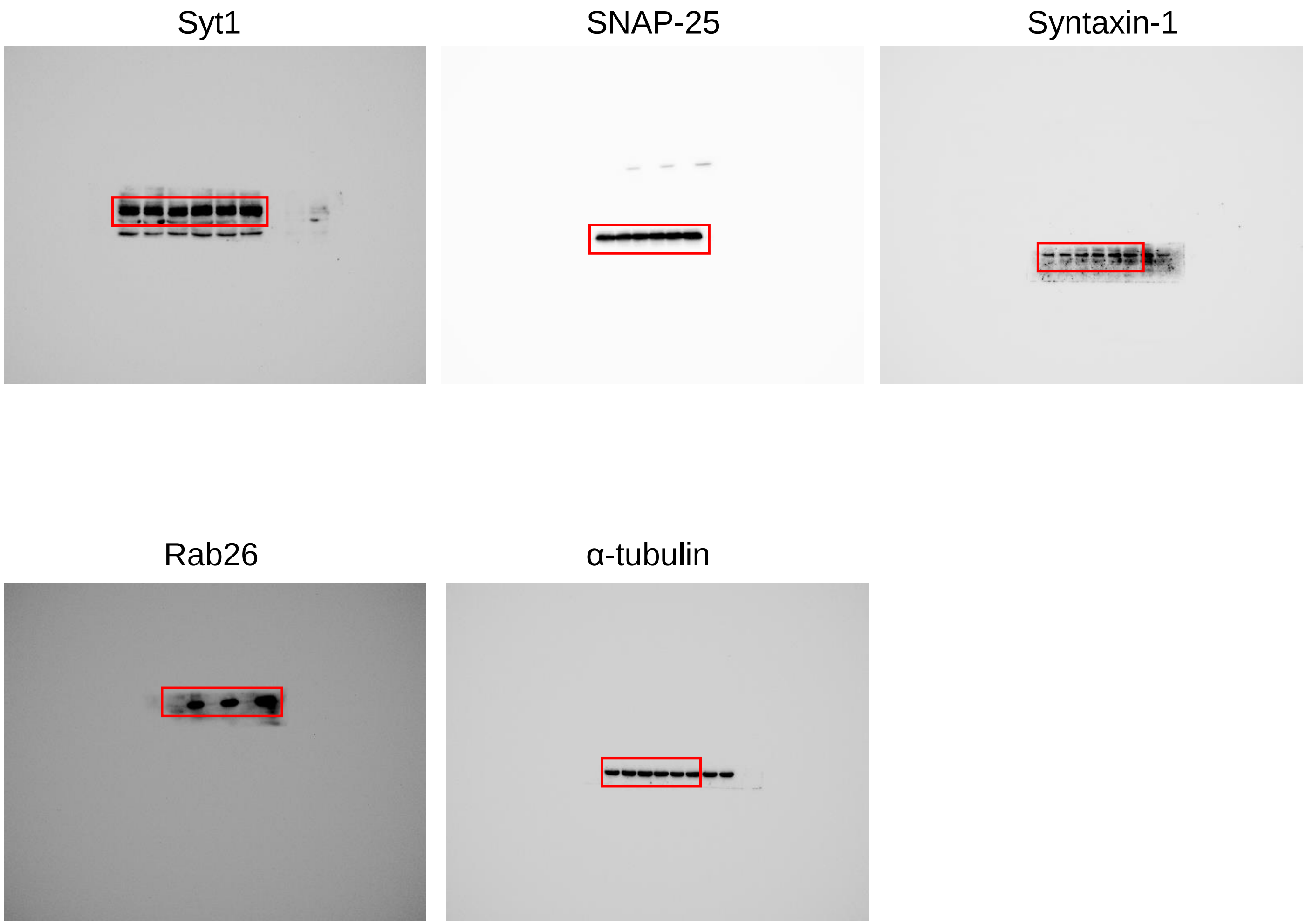

Fig S5A

LC3

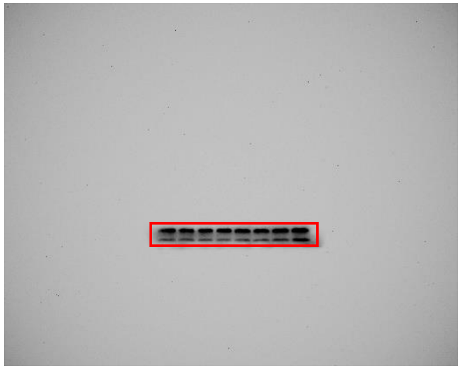

Rab26

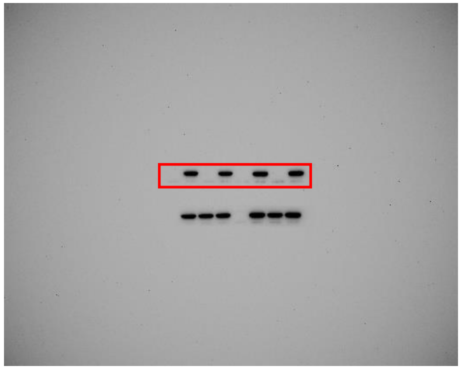

α-tubulin

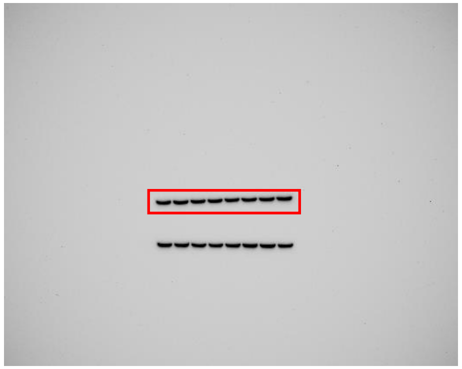

Fig S5C

LC3

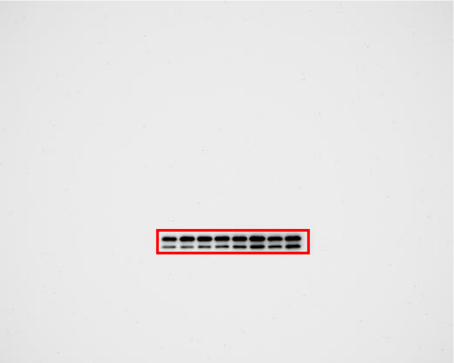

Rab26

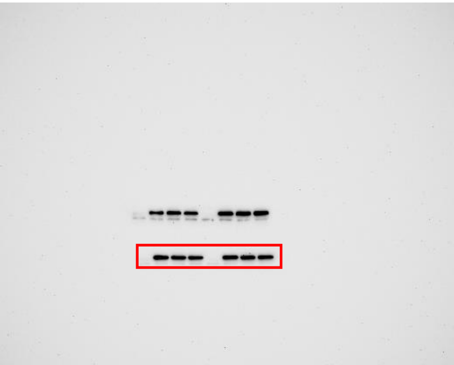

α-tubulin

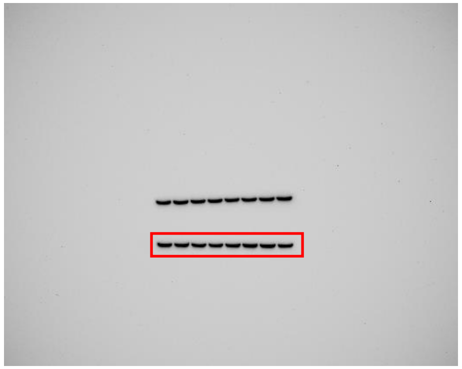

Fig S5E

LC3

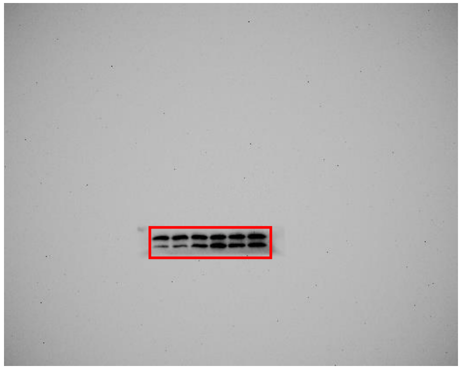

Rab26

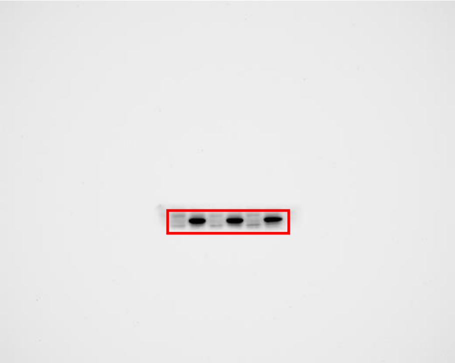

α-tubulin

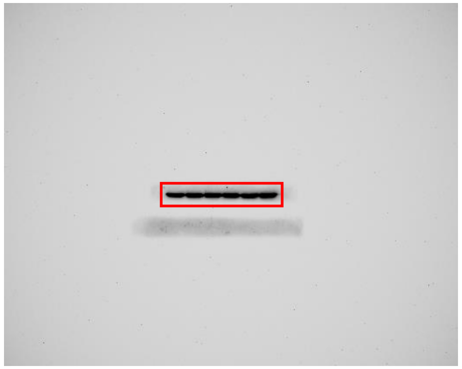

Supplement: S1 Raw Images — (PDF) [file pbio.3002142.s008.pdf]
